# Reduced Oct4 Expression Directs a Robust Pluripotent State with Distinct Signaling Activity and Increased Enhancer Occupancy by Oct4 and Nanog

**DOI:** 10.1016/j.stem.2013.04.023

**Published:** 2013-05-02

**Authors:** Violetta Karwacki-Neisius, Jonathan Göke, Rodrigo Osorno, Florian Halbritter, Jia Hui Ng, Andrea Y. Weiße, Frederick C.K. Wong, Alessia Gagliardi, Nicholas P. Mullin, Nicola Festuccia, Douglas Colby, Simon R. Tomlinson, Huck-Hui Ng, Ian Chambers

**Affiliations:** 1 MRC Centre for Regenerative Medicine, Institute for Stem Cell Research, School of Biological Sciences, University of Edinburgh, 5 Little France Drive, Edinburgh EH16 4UU, Scotland, UK; 2 SyntheSys, Synthetic & Systems Biology, School of Biological Sciences, University of Edinburgh, Mayfield Road, Edinburgh EH9 3JD, Scotland, UK; 3 Gene Regulation Laboratory, Genome Institute of Singapore, Singapore 138672, Singapore; 4 Graduate School for Integrative Sciences and Engineering, Department of Biological Sciences, and Department of Biochemistry, National University of Singapore, Singapore 117597, Singapore; 5 School of Biological Sciences, Nanyang Technological University, Singapore 637551, Singapore

## Abstract

Embryonic stem cell (ESC) pluripotency is governed by a gene regulatory network centered on the transcription factors Oct4 and Nanog. To date, robust self-renewing ESC states have only been obtained through the chemical inhibition of signaling pathways or enforced transgene expression. Here, we show that ESCs with reduced Oct4 expression resulting from heterozygosity also exhibit a stabilized pluripotent state. Despite having reduced Oct4 expression, *Oct4*^+/−^ ESCs show increased genome-wide binding of Oct4, particularly at pluripotency-associated enhancers, homogeneous expression of pluripotency transcription factors, enhanced self-renewal efficiency, and delayed differentiation kinetics. Cells also exhibit increased Wnt expression, enhanced leukemia inhibitory factor (LIF) sensitivity, and reduced responsiveness to fibroblast growth factor. Although they are able to maintain pluripotency in the absence of bone morphogenetic protein, removal of LIF destabilizes pluripotency. Our findings suggest that cells with a reduced Oct4 concentration range are maintained in a robust pluripotent state and that the wild-type Oct4 concentration range enables effective differentiation.

## Highlights

• Reduced Oct4 results in homogeneous transcription factor level and delayed differentiation kinetics
• Oct4^+/−^ ESCs show increased Oct4 and Nanog binding at pluripotency enhancers
• Oct4^+/−^ ESCs exhibit increased Wnt, phospho-STAT3, and LIF sensitivity
• Reduced Oct4 levels robustly maintain ESC pluripotency without BMP/serum/2i

## Teaser

Reduced Oct4 expression leads to increased Oct4 and Nanog binding to key pluripotency network sites, altered signaling responses that favor self-renewal, and a stabilized pluripotent state that inhibits differentiation.

## Introduction

Pluripotency is promoted and stabilized by a pluripotent-cell-specific gene regulatory network (GRN) (Chambers and Tomlinson, 2009; Jaenisch and Young, 2008; Ng and Surani, 2011). Central to this network are the transcription factors Oct4, Sox2, and Nanog. ESCs fluctuate between states of high Nanog expression, possessing high self-renewal efficiency and low Nanog expression, exhibiting increased differentiation propensity (Chambers et al., 2007). Notably, Nanog is also expressed heterogeneously in the preimplantation embryo (Chazaud et al., 2006; Dietrich and Hiiragi, 2007; low Nanog expression facilitates primitive endoderm differentiation, and high Nanog expression maintains epiblast identity. The importance of the Nanog-low state for proper embryonic development is underscored by the finding that embryos expanded from the eight-cell stage in the presence of drugs that inhibit MEK and fail to develop a primitive endoderm (Nichols et al., 2009; Yamanaka et al., 2010); conversely, the supplementation of embryos in culture with fibroblast growth factor (FGF) results in embryos in which epiblast identity is lost in favor of primitive endoderm (Yamanaka et al., 2010). In ESCs, MEK inhibition upregulates Nanog (Silva et al., 2009). How FGF regulates Nanog is unknown, but the process may involve the allelic regulation of Nanog through downstream MEK targets (Miyanari and Torres-Padilla, 2012). In addition to Nanog, other pluripotency factors, such as Esrrb (van den Berg et al., 2008), Rex1 (Toyooka et al., 2008), Stella (Hayashi et al., 2008), Klf4, and Tbx3 (Niwa et al., 2009), are expressed heterogeneously in ESCs. Although the mechanisms generating ESC heterogeneity have begun to be characterized (Fidalgo et al., 2012; Navarro et al., 2012; Villasante et al., 2011), how populations of ESCs expressing homogeneously high levels of transcription factors arise in the presence of serum is not well understood.

Recently, ESCs cultured in the presence of MEK and GSK inhibitors (Ying et al., 2008) have been reported to express a more homogenous level of pluripotency transcription factors (Wray et al., 2010). However, the maintenance of this “ground state” requires a continuous supply of MEK and GSK 3 inhibitors. Therefore, it is of considerable interest to determine whether a robust pluripotent state can exist in the absence of such pharmacological inhibition. Oct4 levels, generally considered to be uniform in undifferentiated ESCs, are critical not only in enabling self-renewal but also for the outcome of differentiation (Niwa et al., 2000). However, these prior studies focused either on the complete elimination or overexpression of Oct4. Here, we demonstrate that Oct4 levels in ESCs cultured in serum occupy a broad unimodal peak; genetic exclusion of the highest Oct4 expressers enables the robust propagation of pluripotency with leukemia inhibitory factor (LIF) as the sole requirement. In this condition, the binding of Oct4 to key regulatory nodes of the pluripotency GRN is enhanced, and signaling responses are tilted in favor of self-renewal.

## Results

### *Oct4*^*+/−*^ Cells Express Increased Nanog and Have a Reduced Nanog-Negative Population

**Figure 1:**
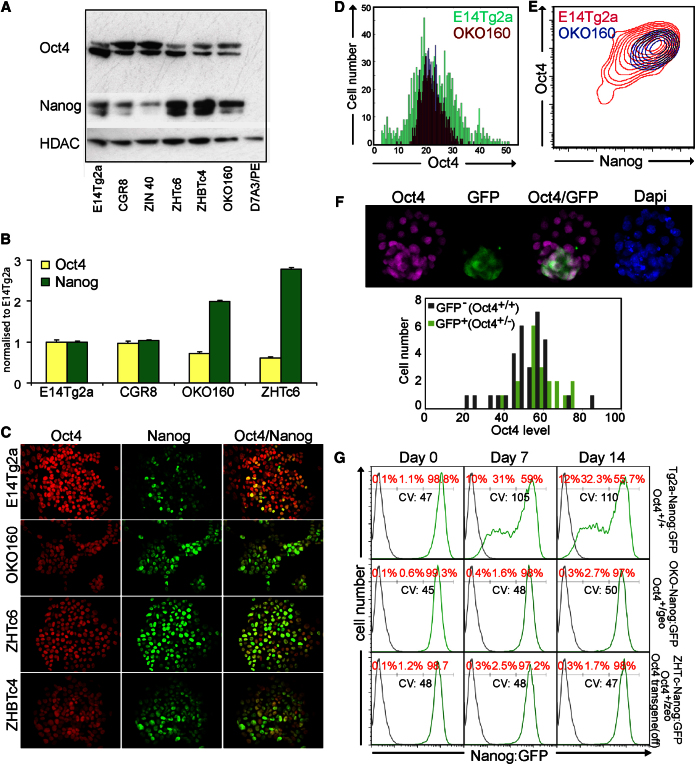
*Oct4*^*+/−*^ Cells Express an Elevated Level of NANOG and Lack a Nanog-Negative Population (A) Immunoblot analysis of *Oct4*^*+/+*^ (E14Tg2a, CGR8, and ZIN40) and of *Oct4*^*+/−*^ (OKO160 [Mountford et al., 1998] and ZHTc6 [cultured in 1 μg/ml doxycycline [Niwa et al., 2000]) cell lines. See Table S1 for a detailed summary of all cell lines used in this study. ZHBTc4 (*Oct4*^*−/−*^) cells (Niwa et al., 2000), which express an Oct4 transgene at heterozygous levels (when cultured without doxycycline) also showed increased Nanog. The parietal endoderm cell line D7A3/PE (Dani et al., 1998) provides a negative control. (B) Quantitative PCR (qPCR) analysis of Oct4 and Nanog mRNAs in *Oct4*^*+/+*^ (E14Tg2a and CGR8) and *Oct4*^*+/−*^ (OKO160 and ZHTc6 [cultured in 1 μg/ml doxycycline]) cells. Error bars represent SD; n = 3. (C) Immunofluorescence analysis of wild-type (WT; E14Tg2a) and Oct4 mutant (OKO160, ZHTc6 [cultured in 1 μg/ml doxycycline], and ZHBTc4 [cultured without doxycycline]) cells for Nanog (green) and Oct4 (red). (D) Quantitative analysis of Oct4 protein levels in individual cells in colonies of *Oct4*^*+/+*^ (E14Tg2a, green) and *Oct4*^*+/−*^ (OKO160, brown) cells. (E) Intracellular FACS quantitation of Oct4 and Nanog protein levels in individual cells of *Oct4*^*+/+*^ (E14Tg2a, red) and *Oct4*^*+/−*^ (OKO160, blue) cultures. (F) Top, immunofluorescence analysis of blastocyst expression of Oct4 protein following the aggregation of GFP-marked *Oct4*^*+/−*^ ESCs with WT morula. Bottom, quantitation of immunofluorescence for Oct4 in the ICM for WT and GFP-marked *Oct4*^*+/−*^ cells. (G) FACS analysis for Nanog:GFP in Oct4 WT (Tg2a-Nanog:GFP) and Oct4 mutant cells (OKO-Nanog:GFP and ZHTc-Nanog:GFP [cultured in 1 μg/ml doxycycline] derived from the parental lines OKO160 and ZHTc6, respectively; Oct4 genotypes are indicated) at the indicated times following release from puromycin selection (day 0). The percentage of cells in Nanog-low, Nanog-middle, and Nanog-high populations are shown (red) with the coefficient of variation (CV) for Nanog:GFP indicated. See also Figure S1 and Tables S1 and S2.

Global chromatin localization (Chen et al., 2008; Kim et al., 2008; Loh et al., 2006; Marson et al., 2008) and transient expression assays (Kuroda et al., 2005; Rodda et al., 2005) suggest that Oct4 positively regulates *Nanog* gene expression. A clear prediction from this is that cells that have reduced Oct4 levels should have reduced Nanog levels. Therefore, it was with surprise that we noted that multiple ESC lines in which homologous recombination had introduced a drug-resistance gene trap cassette to the *Oct4* locus, expressed elevated levels of Nanog protein and messenger RNA (mRNA) when compared to *Oct4*^+/+^ ESCs (Figures 1A and 1B; see Table S1 [available online] for a list of cell lines used in this study). Previously, Nanog has been demonstrated to be expressed heterogeneously in undifferentiated ESCs (Chambers et al., 2007; Hatano et al., 2005; Singh et al., 2007). Therefore, this increase in Nanog protein could be due to either a general increase in the level of Nanog per cell or an alteration in the proportion of cells in the Nanog-negative compartment. Immunofluorescence analysis showed that *Oct4*^+/−^ ESCs expressed Nanog more uniformly throughout the colonies than *Oct4*^+/+^ ESCs and at levels comparable to those present in Nanog-high nuclei in *Oct4*^+/+^ colonies (Figure 1C).

Although Oct4 protein has been reported to be expressed at 65% wild-type (WT) levels in populations of *Oct4*^+/−^ ESCs by immunoblot analysis (Niwa et al., 2000), the range of expression in individual cells has not been addressed. Immunofluorescence analysis (Figure 1D) shows that the range of Oct4 levels in individual *Oct4*^+/−^ cells strongly overlaps the distribution of Oct4 levels seen in WT cells. Not only do *Oct4*^+/−^ ESC colonies have a reduced number of cells that express high levels of Oct4 (Figure 1D), but, interestingly, cells that lack Oct4 are also missing (Figure 1D), a point we return to below. Moreover, aggregation of *Oct4*^*+/−*^ ESCs with WT embryos showed that the Oct4 protein levels in *Oct4*^*+/−*^ cells were within the range observed in WT inner cell mass (ICM) cells (Figure 1F). Intracellular fluorescence-activated cell sorting (FACS) analysis in bulk ESC cultures showed that the levels of both Oct4 and Nanog present in individual *Oct4*^+/−^ ESCs are within the normal range found in WT ESCs (Figure 1E). In addition, although there is no effect of altering the Nanog dosage upon the range of Oct4 expression levels, as recently reported (Navarro et al., 2012), reducing Oct4 to the level present in *Oct4*^+/−^ ESCs shifts the level of Nanog to the higher end of the range detected in WT ESCs. Altogether, these results indicate that the increase in Nanog expression in *Oct4*^+/−^ cell lines is due to a reduction in the proportion of Nanog-low and Nanog-negative cells.

To examine dynamic aspects of heterogeneous transcription factor regulation, the previously described targeting vector conferring *Nanog*-directed expression of green fluorescent protein (GFP) and puromycin resistance (Chambers et al., 2007) was introduced into two different Oct4 mutant cell lines via homologous recombination (Figures S1A and S1B). The temporal response to puromycin withdrawal confirmed that *Oct4*^+/−^ ESCs retain homogenous GFP expression under conditions in which typically heterogeneous GFP expression emerges in WT cultures (Figure 1G).

### Titrated Elevation of Oct4 Levels Restores the Heterogeneous Expression of Nanog, Esrrb, and Klf4

**Figure 2:**
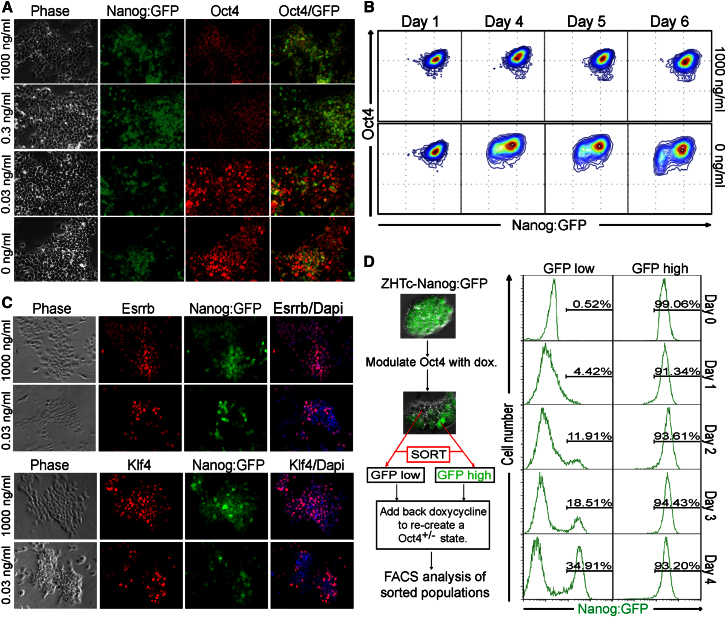
Titrated Elevation of Oct4 Levels Restores the Heterogeneous Expression of Nanog, Esrrb, and Klf4 (A) Immunofluorescence (Oct4, red) and fluorescence (Nanog:GFP, green) in ZHTc-Nanog:GFP cells treated with the indicated doxycycline concentrations for 6 days. (B) Intracellular FACS analysis for Oct4 and Nanog:GFP at the indicated times of treatment with the indicated doxycycline dose. (C) Immunofluorescence analysis at day 4 of doxycycline treatment of ZHTc-Nanog:GFP cells for Esrrb, Klf4, and Nanog:GFP. (D) GFP-low ZHTc-Nanog:GFP cells remain undifferentiated and revert to a GFP-expressing state. Left, an experimental scheme; Oct4 expression was increased in *Oct4*^*+/−*^ (ZHTc-Nanog:GFP) cells in order to induce Nanog heterogeneity, and cells were sorted into Nanog:GFP-high and Nanog:GFP-low populations. Right, reanalysis of the sorted populations showed that they were >99% pure (day 0). Cells were replated in GMEMβ-FCS-LIF-doxycycline (1,000 ng/ml) in order to restore Oct4 protein to a *Oct4*^*+/−*^ state and the emergence of the GFP-high population (indicated as a percentage) monitored daily. See also Figure S2 and Table S1.

The correlation between homogeneous Nanog expression and *Oct4* heterozygosity in independent cell lines suggests a causative role for the Oct4 protein level in the generation of Nanog heterogeneity. To test this hypothesis, the effect of increasing the Oct4 level was examined with ZHTc-Nanog:GFP cells (Figures S1A and S1 B and Table S1). In addition to the Nanog:GFP reporter, these cells also contain a doxycycline-suppressible Oct4 transgene. If homogeneous Nanog expression was caused by reduced Oct4 levels, then raising the Oct4 level by titrating down the doxycycline concentration should restore Nanog heterogeneity. This was investigated by immunofluorescence (Figure 2A) and intracellular FACS (Figures 2B, S2A, and S2B). No changes in Oct4 or Nanog were seen during the first 2 days; however, by day 3, increasing Oct4 expression was detected at doxycycline concentrations of 0.3 ng/ml or less (Figure S2A). Interestingly, the level of Oct4 expression obtained with 0.03 ng/ml doxycycline approached, but did not exceed, the level observed in the *Oct4*^+/+^ control line E14Tg2a-Nanog:GFP. Decreased Nanog expression also became detectable by day 3, and the population resolved into a bimodal expression pattern by day 4 (Figures S2A and S2B). Importantly, bimodal Nanog expression was achieved at the doxycycline concentration (0.03 ng/ml) that elevated Oct4 toward WT levels and did not require Oct4 overexpression (Figures S2A and 2A). The restoration of Nanog heterogeneity was reflected by the increased coefficient of variation of Nanog:GFP at doxycycline concentrations of 0.1 ng/ml or less (Figure S2B). These results indicate that the lack of Nanog-low and Oct4-low cells in *Oct4*^+/−^ cultures is not due to aberrant selection for cells with an enhanced self-renewal propensity but is a consequence of the Oct4 expression level; restoring the Oct4 level toward, but not beyond, WT levels is sufficient to restore Nanog heterogeneity. Dynamically, the increase in Oct4 level results in decreased Nanog expression in a subpopulation of cells, and, subsequently, Oct4-low cells emerge from this Nanog-low population (Figure 2B).

The Nanog-low cells formed by doxycycline treatment of the ZHTc-Nanog:GFP cells expressed high SSEA1^+^, suggesting that they were undifferentiated (Figure S2C). Nevertheless, the question remained as to whether Nanog-low cells induced in ZHTc-Nanog:GFP cultures after Oct4 upregulation were already committed to differentiate. Therefore, as schematized in Figure 2D, SSEA1^+^-GFP^−^ cells were sorted from ZHTc-Nanog:GFP cells cultured in reduced doxycycline and replated in culture with 1 μg/ml doxycycline. By day 4, 35% of the sorted GFP^−^ cells had reverted to a GFP^+^ state (Figure 2D). This not only indicates that GFP^−^ cells can remain undifferentiated but also confirms the crucial role of Oct4 in facilitating switching both from Nanog-high to Nanog-low states and also from Nanog-low to Nanog-high states.

Immunofluorescence analysis showed that, similar to Nanog, Esrrb and Klf4 were expressed relatively homogeneously in *Oct4*^+/−^ cultures (Figure S1C). Upon the restoration of Oct4 toward WT levels, both Esrrb and Klf4 became heterogeneously expressed (Figure 2C). Therefore, these findings identify Oct4 as a critical facilitator of the switch between heterogeneous and homogeneous states of transcription factor expression in ESCs.

### ESCs Expressing Reduced Oct4 Levels Show Delayed Differentiation

**Figure 3:**
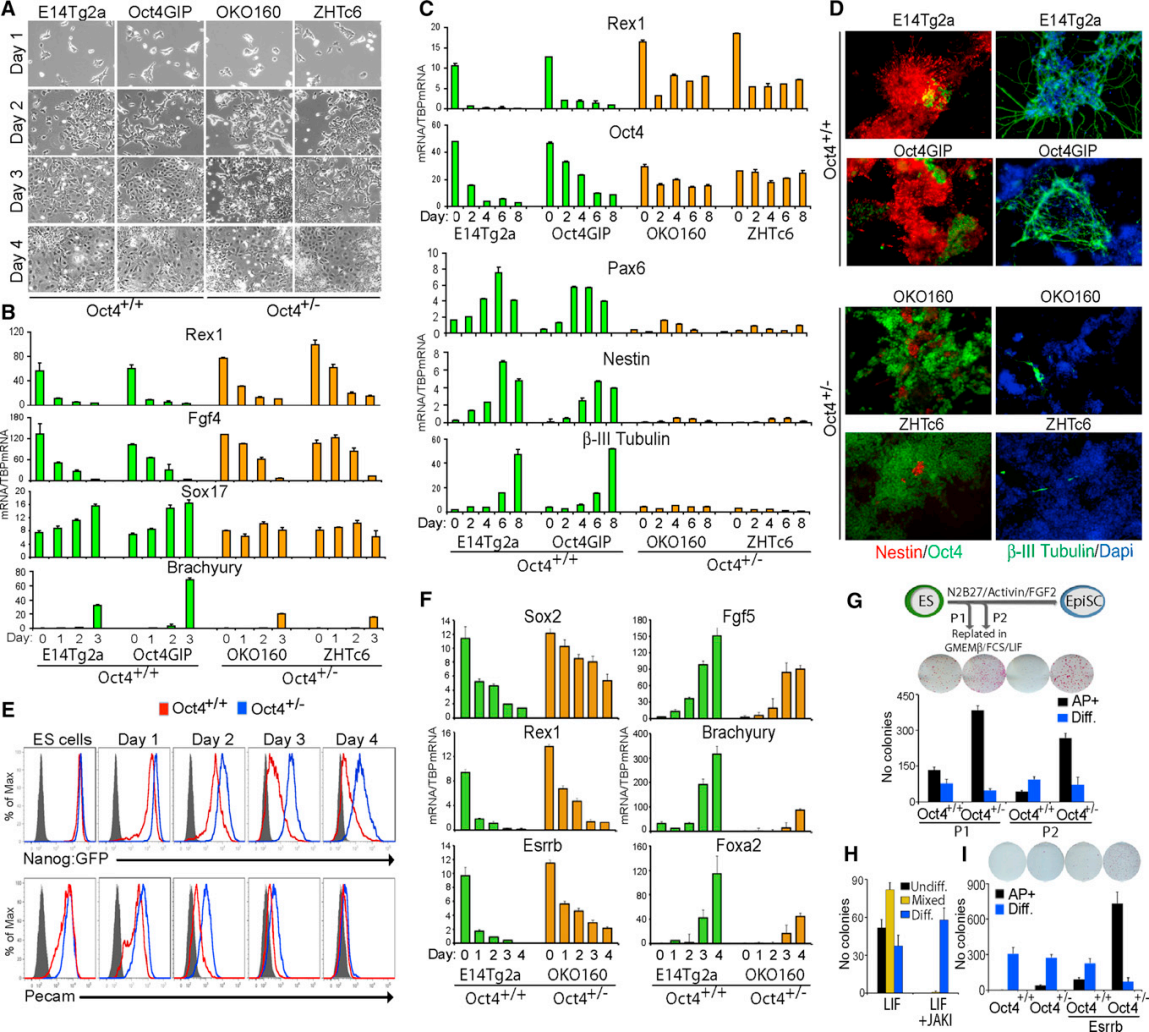
ESCs Expressing Reduced Oct4 Levels Show Delayed Differentiation (A) Morphology of *Oct4*^*+/+*^ (E14Tg2a and Oct4GiP [which carries an Oct4 promoter-driven GFP-ires-pac-pA cassette as an additive transgene]) (Ying et al., 2002) and *Oct4*^+/−^ (OKO160 and ZHTc6) cell lines at the indicated times after LIF withdrawal. (B) qPCR analysis of mRNA expression in the indicated cell lines on the days shown after LIF withdrawal. Error bars represent SD; n = 3. (C) qPCR analysis of mRNA expression in the indicated cell lines on the days shown after the initiation of monolayer neural differentiation. Error bars represent SD; n = 3. (D) Immunofluorescence for Nestin, Oct4, or βIII Tubulin at day 8 of neural differentiation. Cells were counterstained with DAPI. (E) FACS analysis of Nanog:GFP and Pecam expression after the replating of *Oct4*^*+/+*^ (Tg2a-Nanog:GFP) and *Oct4*^*+/−*^ (OKO-Nanog:GFP) ESCs in activin-FGF for the allowance of differentiation to an EpiSC state. (F) qPCR analysis of mRNA expression in the indicated cell lines on the days shown after the replating of ESCs in activin-FGF2. Error bars represent SD; n = 3. (G) Functional assessment of differentiation status. Following the outlined scheme (top), the differentiation status of ES-like colonies was assessed after 6 days (*Oct4*^*+/+*^, E14Tg2a; *Oct4*^*+/−*^, OKO160). Error bars represent SD; n = 4. (H) JAK inhibitor blocks ESC self-renewal in the presence of LIF. ESCs were treated with JAK inhibitor for 6 days, replated at clonal density without JAK inhibitor in GMEMβ-FCS-LIF, cultured for an additional 6 days, and alkaline phosphatase-positive counted. Error bars represent SD; n = 3. (I) EpiSC lines of the indicated genotypes (*Oct4*^*+/+*^, E14Tg2a; *Oct4*^*+/−*^, OKO160) were passaged twice in the presence of the same JAK inhibitor concentration used in (H), replated in GMEMβ-FCS-LIF either in the presence or the absence of exogenous Esrrb, and assessed for colony-forming capacity after 6 days. Error bars represent SD; n = 4. See also Figures S3 and S4 and Tables S1 and S2.

Oct4 has previously been shown to be necessary for ESC self-renewal, given that the elimination of Oct4 results in trophectodermal differentiation, whereas overexpression causes a proportion of cells to differentiate into cells expressing endoderm and mesoderm markers (Niwa et al., 2000). How Oct4 mediates this differentiation remains unknown. A priori, it might be expected that *Oct4*^+/−^ cells would differentiate more rapidly. However, the foregoing results showing that *Oct4*^+/−^ cells have a reduced proportion of Nanog-negative cells, along with the model that differentiation occurs more effectively through Nanog-low cells (Chambers et al., 2007), prompted an investigation of this point. Upon LIF withdrawal, *Oct4*^+/−^ cell lines showed delayed signs of morphological differentiation (Figure 3A), delayed downregulation of the pluripotency markers Rex1 and FGF4, and reduced upregulation of the differentiation markers Brachyury and Sox17 (Figure 3B) in comparison to *Oct4*^+/+^ ESCs.

Neural differentiation (Ying et al., 2008) was also delayed in *Oct4*^+/−^ ESCs (Figures 3C and 3D). The persistence of large numbers of Oct4-expressing cells in *Oct4*^+/−^ cultures (Figure 3D) indicates that these cells were unable to undergo efficient neural differentiation not because they were being diverted into nonneural lineages but because they remained undifferentiated.

Next, the ability of *Oct4*^+/−^ ESCs to differentiate into epiblast stem cells (EpiSCs) was investigated by replating *Oct4*^+/−^ and *Oct4*^+/+^ ESCs in activin and FGF. *Oct4*^+/−^ ESCs showed a slower reduction in the expression of Nanog:GFP, Pecam (Figure 3E), and ESC-specific transcripts and a delayed, diminished induction of EpiSC transcripts (Figure 3F). To functionally analyze commitment, we replated cells into GMEMβ-FCS-LIF after one or two passages in N2B27-activin-FGF2. Cultures of *Oct4*^+/−^ cells retain a greater ability to form alkaline phosphatase-positive undifferentiated ESC colonies than *Oct4*^+/+^ cultures (Figure 3G). These results indicate that *Oct4*^+/−^ ESCs are compromised in their ability to efficiently commit toward differentiation and that this delay is in the initial exit from the ESC state.

Nevertheless, EpiSC lines could be established from *Oct4*^+/−^ ESCs by six passages in activin-FGF. Once established, cells were passaged for an additional 2 weeks in the presence of a JAK inhibitor in order to ensure the complete elimination of any residual ESCs; effective JAK inhibition was confirmed with ESCs (Figure 3H). Despite prior JAK inhibition, *Oct4*^+/−^ EpiSCs were able to form occasional colonies when replated in GMEMβ-FCS-LIF (Figure 3I), suggesting that, in the presence of reduced Oct4 protein, the stringent separation between ESC and EpiSC states is compromised. *Oct4*^+/−^ EpiSCs also displayed a greater propensity to generate induced pluripotent stem cells than WT cells, as shown by the increased colony number formed after the expression of Esrrb (Festuccia et al., 2012) and prior to replating in GMEMβ-FCS-LIF (Figure 3I).

### Restoration of Nanog Heterogeneity in *Oct4*^+/−^ Cells Rescues Retarded Differentiation

Our previous studies suggested that differentiation proceeds efficiently after transient downregulation of Nanog (Chambers et al., 2007). This predicts that the retarded differentiation kinetics of the *Oct4*^+/−^ cells could be rescued by restoring a Nanog-low compartment to the population. Therefore, we used ZHTc-Nanog:GFP cells to vary the Oct4 level (as outlined in Figure S3A) and examined differentiation kinetics. Transient treatment of ZHTc-Nanog:GFP cells for 4 days with the doxycycline concentration shown in order to elevate Oct4 expression toward WT levels (0.03 ng/ml; Figure S2A) produced a decrease in Nanog mRNA (Figure S3B) and the heterogeneous expression of Nanog:GFP (Figure S3A). Continued induction of the Oct4 transgene during the differentiation assays was prevented by reapplying doxycycline at 1 μg/ml. After 8 days in the neural differentiation protocol, cells in which heterogeneous Nanog expression was initially imposed showed increased expression of neural markers in comparison to cells maintained in 1 μg/ml doxycycline (Figures S3C and S3D). The response of cells to LIF withdrawal was also assessed. Cells in which heterogeneous Nanog expression was initially induced showed a more differentiated morphology (Figure S3E) and an enhanced expression of differentiation markers (Figure S3F) in comparison to cells maintained in 1 μg/ml doxycycline. These results indicate that the slower differentiation of *Oct4*^+/−^ cells is attributable to a lack of heterogeneous Nanog expression and that this can be rescued by restoring the full range of Oct4 levels observed in WT ESCs.

### Oct4-Induced Differentiation Is Blocked by Nanog

The above results suggest that differentiation induced by Oct4 elevation occurs via production of a differentiation-prone Nanog-low population. If so, differentiation should be blocked by enforced Nanog expression. This was tested with episomally supertransfectable cells (Chambers et al., 2003) carrying a loxP-flanked Nanog transgene (Nanog/T cells). In comparison to control cells, which differentiated upon Oct4 transfection (Figure S4A), Nanog/T cells formed morphologically undifferentiated colonies (Figure S4A). To establish that this differentiation blockade was due to transgenic Nanog expression, we deleted the loxP-flanked Nanog ORF by Cre transfection. Cre-mediated Nanog excision results in CAG-driven GFP expression. Although most green colonies formed by Cre treatment of empty vector transfectants had an undifferentiated morphology (Figures S4B and S4C), the majority of green colonies derived from Oct4 transfectants were completely differentiated (Figures S4B and S4C). Therefore, Oct4 induces differentiation by the production of a Nanog-low cell population.

### *Oct4*^+/−^ Cells Exhibit Enhanced Clonal Self-Renewal Properties

**Figure 4:**
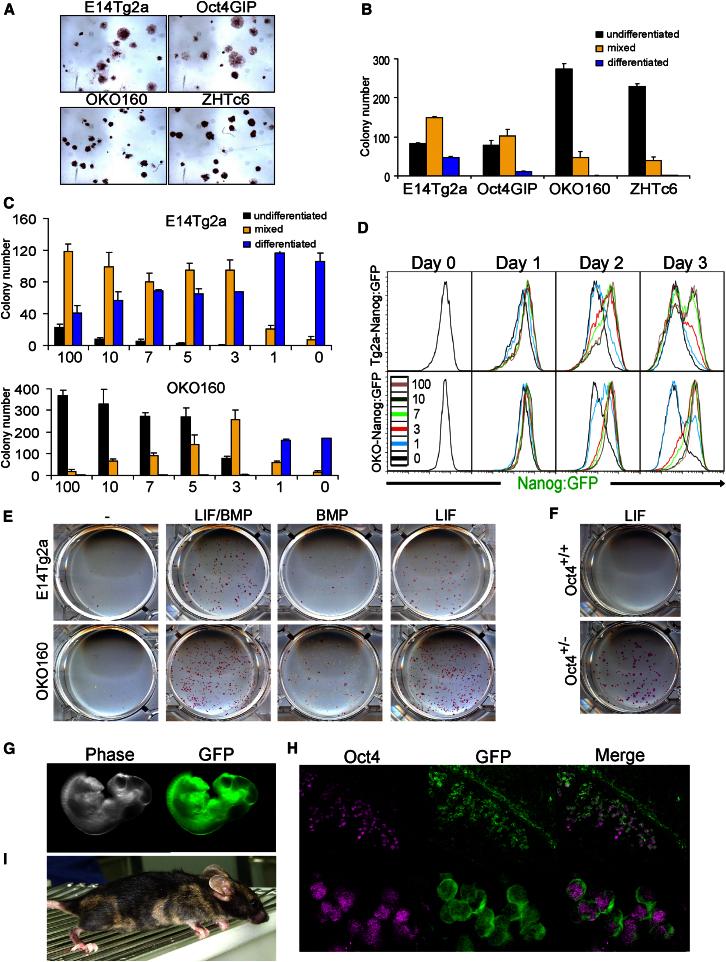
Pluripotency of *Oct4*^+/−^ Cells Is Supported in the Absence of BMP (A) Alkaline phosphatase staining of colonies formed by plating the indicated cell lines at clonal density and culturing for 7 days in GMEMβ with 10% FCS and 100 U/ml LIF. (B) Quantitation of colony types formed by cell lines shown in (A). Error bars represent SD; n = 3. (C) Quantitation of colony types formed by plating *Oct4*^+/+^ (E14Tg2a) and *Oct4*^+/−^ (OKO160) ESCs at clonal density in GMEMβ with 10% FCS and the indicated LIF concentrations (U/ml) for 7 days. Error bars represent SD; n = 3. (D) FACS analysis of Tg2a-Nanog:GFP and OKO-Nanog:GFP cells treated with the indicated LIF concentrations (U/ml) for 3 days. Before starting (day 0), cell lines were selected for Nanog:GFP expression with puromycin. (E) *Oct4*^*+/+*^ (E14Tg2a) or *Oct4*^*+/−*^ ESCs (OKO160-GFP) were plated at clonal density, cultured in N2B27 (with the indicated cytokines) for 7 days, and stained for alkaline phosphatase. (F) *Oct4*^*+/+*^ (AGFP7) or *Oct4*^*+/−*^ (OKO160-GFP) ESCs were cultured in N2B27-LIF (100 U/ml) for 7 days at clonal density, replated for a further 7 days in the same conditions, and stained for alkaline phosphatase. (G) *Oct4*^*+/−*^ ESCs (OKO160-GFP, which express a constitutive GFP transgene) cultured as described in (F) were injected into a host blastocyst and midgestation embryos examined for the presence of ubiquitous GFP-expressing *Oct4*^+/−^ cells by fluorescence microscopy. (H) *Oct4*^*+/−*^ ESCs (OKO-AGFP, which express a constitutive GFP transgene) were treated as described in (F) and (G). E11.5 genital ridges were analyzed by immunofluorescence for Oct4 protein and GFP expression. (I) Adult chimera from a blastocyst obtained as described in (G).

In comparison to *Oct4*^+/+^ cell lines, clonal self-renewal assays show that independent *Oct4*^+/−^ ESC lines have an enhanced ability to form uniformly undifferentiated colonies in response to saturating LIF concentrations (Figures 4A and 4B). Further clonal self-renewal assays showed that *Oct4*^+/−^ ESCs have ∼30-fold greater sensitivity to LIF (Figure 4C). FACS analysis of the effect of LIF concentration on Nanog expression showed that, although Nanog was heterogeneous in WT cells even at the highest LIF concentration, 10 U/ml LIF was sufficient to maintain a unimodally high Nanog:GFP profile in *Oct4*^+/−^ ESCs (Figure 4D). These results indicate that *Oct4*^+/−^ ESCs show enhanced responsiveness to LIF stimulation.

In N2B27 supplemented with LIF and bone morphogenetic protein (BMP), both *Oct4*^+/+^ and *Oct4*^+/−^ cells formed self-renewing colonies, whereas, in N2B27 alone or in N2B27 supplemented with BMP alone, the formation of undifferentiated colonies was severely impaired (Figure 4E). This was also true for WT cells plated in the presence of LIF alone. In contrast, *Oct4*^+/−^ cells formed alkaline phosphatase-positive undifferentiated colonies in LIF alone (Figure 4E).

### Pluripotency of *Oct4*^+/−^ Cells Is Supported in the Absence of BMP/Serum/2i

To determine whether pluripotency could be sustained during the clonal propagation of *Oct4*^+/−^ cells in LIF alone, we obtained derivative cells expressing GFP constitutively. These cells were plated at clonal density, expanded, and replated at clonal density in defined medium in recombinant LIF without BMP. After two passages, parental cells completely differentiated and could not be sustained (Figure 4F). In contrast, *Oct4*^+/−^ ESCs efficiently formed uniformly alkaline phosphatase-positive undifferentiated colonies (Figure 4F). Similar results were obtained when Oct4 was targeted in AGFP ESCs (Gilchrist et al., 2003). When GFP^+^:*Oct4*^+/−^ ESCs were injected into the blastocoel cavity of a host C57Bl/6 host embryo, midgestation embryos were obtained in which GFP^+^:*Oct4*^+/−^ cells were widely distributed (Figure 4G) and detectable in the germline at E11.5 (Figure 4H). When similarly injected embryos were allowed to develop to term, adult coat color chimeras were obtained (Figure 4I). Altogether, these data indicate that ESCs with reduced Oct4 levels are released from BMP dependence.

### Oct4 Action on *Nanog*

**Figure 5:**
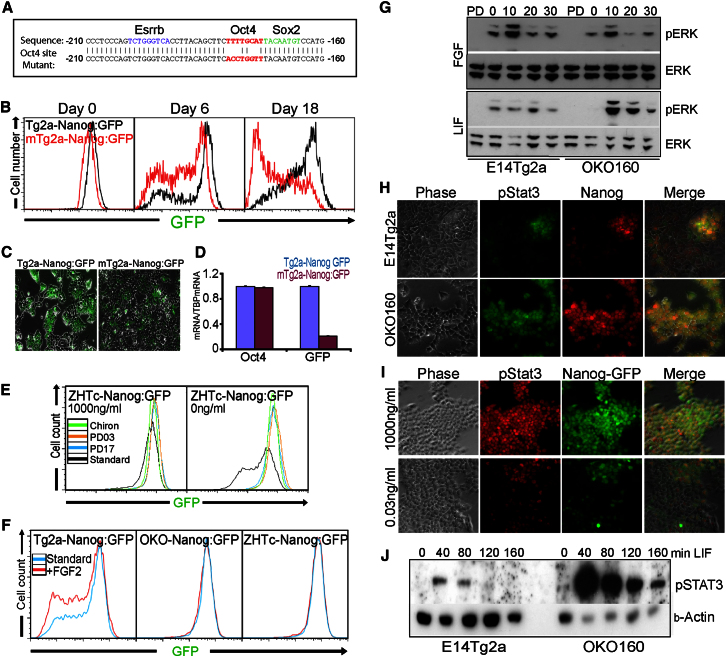
Signaling Influences in *Oct4*^+/−^ ESCs (A) The *Nanog* promoter sequence around the octamer site (red). Binding sites for Essrb (blue) and Sox2 (green) are also shown as is the sequence of the Oct4 site mutation (red). (B and C) Analysis of Tg2a-Nanog:GFP and a representative cell line containing a mutated Oct4 binding site (mTg2a-Nanog:GFP) after release from puromycin treatment by FACS at days 6 and 18 (B) and fluorescent microscopy at day 18 (C). (D) qPCR analysis of Oct4 and GFP transcripts in Tg2a-Nanog:GFP and mTg2a-Nanog:GFP cells at day 18 after puromycin removal. Error bars represent SD; n = 3. (E) FACS analysis of ZHTc-Nanog:GFP treated with or without doxycycline and in parallel with FGFR inhibitor (PD173074), MEK inhibitor (PD0325901), or GSK3 inhibitor (CHIR99021) for 6 days. (F) FACS analysis of the indicated Nanog:GFP cell lines treated with or without FGF2 (10 ng/ml) for 6 days. (G) Immunoblot analysis of phospho-ERK and total ERK in the indicated cell lysates prepared after overnight removal of FCS-LIF and stimulation with FGF2 (10 ng/ml) or LIF (100 U/ml) for the indicated time (min); PD indicates ESCs treated with PD0325901 for 4 hr. (H) Immunofluorescence analysis for phospho-STAT3 and Nanog in the indicated cell lines. (I) Immunofluorescence analysis for phospho-STAT3 and Nanog in ZHTc-Nanog:GFP cells after treatment with the indicated doxycycline concentrations. (J) Immunoblot analysis of phospho-STAT3 in the indicated cell lysates prepared after overnight removal of LIF and stimulation with LIF (100 U/ml) for the indicated time. See also Figure S5 and Tables S1 and S2.

Oct4 binds to an octamer consensus site centered −176 bp upstream of the *Nanog* transcription initiation site (Chambers, 2004; Loh et al., 2006) that has been shown to positively regulate Nanog in transient reporter assays (Kuroda et al., 2005; Rodda et al., 2005). However, given that no data exist regarding the action of Oct4 in the context of the endogenous *Nanog* locus, the parsimonious hypothesis that Oct4 suppresses *Nanog* via the −176 bp octamer site was tested. Gene targeting introduced a mutant version of the GFP-ires-pac-pA vector in which the octamer site in the *Nanog* promoter was destroyed (Figure 5A), thus producing mTg2a-Nanog:GFP ESCs (Figures S5A and S5B). In puromycin, cells carrying the Oct site mutation show a slight shift to lower GFP levels (Figure 5B). Removing puromycin from mTg2a-Nanog:GFP cultures resulted in the increased emergence of GFP-negative cells in comparison to Tg2a-Nanog:GFP cells (Figures 5B and 5C). These effects, mirrored at the mRNA level (Figure 5D), indicate that Oct4 regulates the endogenous *Nanog* locus via the −176 bp site positively, not negatively.

### Altered Signaling Responses in *Oct4*^+/−^ ESCs

Thus far, the results suggest that *Oct4*^+/−^ cells exist in a state of enhanced stability that shares features of the naive ground state (Wray et al., 2010; Ying et al., 2008). To examine the potential connections between the *Oct4*^+/−^ state and the ground state, we induced Oct4 in ZHTc-Nanog:GFP cells in the presence of inhibitors of ERK and GSK3. The induction of Nanog heterogeneity by Oct4 was blocked by treatment with inhibitors of FGFR, MEK, or GSK3 (Figure 5E). These data suggest that elevating Oct4 from heterozygous levels induces heterogeneous Nanog expression by modulating signaling responses.

Fgf4 signaling is implicated in the downregulation of *Nanog* in ESCs (Hamazaki et al., 2006; Kunath et al., 2007) and in the induction of heterogeneity in the ICM (Nichols et al., 2009; Yamanaka et al., 2010). Consistent with this, adding recombinant FGF to Tg2a-Nanog:GFP ESCs increased the proportion of Nanog:GFP-low cells in the culture (Figure 5F). Given that Fgf4 is regulated by Oct4 (Ambrosetti et al., 1997; Yuan et al., 1996), the lack of Nanog heterogeneity in *Oct4*^+/−^ ESCs could be due to reduced Fgf4 or altered Fgf receptor expression by *Oct4*^+/−^ ESCs. However, FGF4 transcripts were elevated in *Oct4*^+/−^ ESCs with no difference detectable in Fgfr2 transcript level (Figure S5C). To further assess the dynamic effects of FGF, we targeted GFP-ires-pac-pA to *Nanog* (Chambers et al., 2007) in *Fgf4*^−/−^ ESCs (Figure S5D). FGF-Nanog:GFP ESCs express Nanog:GFP unimodally (Figure S5E), Nanog:GFP-low cells being induced by FGF addition (Figure S5E). However, adding FGF to parallel cultures of either OKO-Nanog:GFP or ZHTc-Nanog:GFP ESCs did not affect Nanog:GFP, suggesting that the lack of heterogeneity in *Oct4*^+/−^ ESCs is due to an inability of *Oct4*^+/−^ ESCs to respond appropriately to FGF (Figure 5F). To determine whether this lack of response was due to an inability of *Oct4*^+/−^ ESCs to elicit early events downstream of FGF receptor, we examined the kinetics of ERK phosphorylation. When ESCs were cultured in the absence of serum or cytokines and stimulated with FGF, both *Oct4*^+/+^ and *Oct4*^+/−^ ESCs showed a comparable profile and duration of ERK phosphorylation (Figure 5G).

In contrast to FGF stimulation, LIF stimulation increased phospho-ERK more strongly in *Oct4*^+/−^ ESCs (Figure 5G). Phospho-STAT3 was detected heterogeneously in *Oct4*^+/+^ ESCs cultured in 100 U/ml LIF and predominantly in Nanog-expressing cells (Figure 5H). In contrast, almost all *Oct4*^+/−^ cells express phospho-STAT3 (Figure 5H). Moreover, phospho-STAT3 became heterogeneous when ZHTc-Nanog:GFP cells were shifted from 1,000 ng/ml to 0.03 ng/ml doxycycline (Figure 5I). Furthermore, when parallel cultures of *Oct4*^+/+^ and *Oct4*^+/−^ ESCs were starved and restimulated with LIF, phospho-STAT3 was induced to a far higher level in *Oct4*^+/−^ ESCs (Figure 5J). These data suggest that the difference in transcription factor heterogeneity in *Oct4*^+/−^ ESCs may be due to enhanced LIF sensitivity mediated by increased phospho-STAT3.

**Figure 6:**
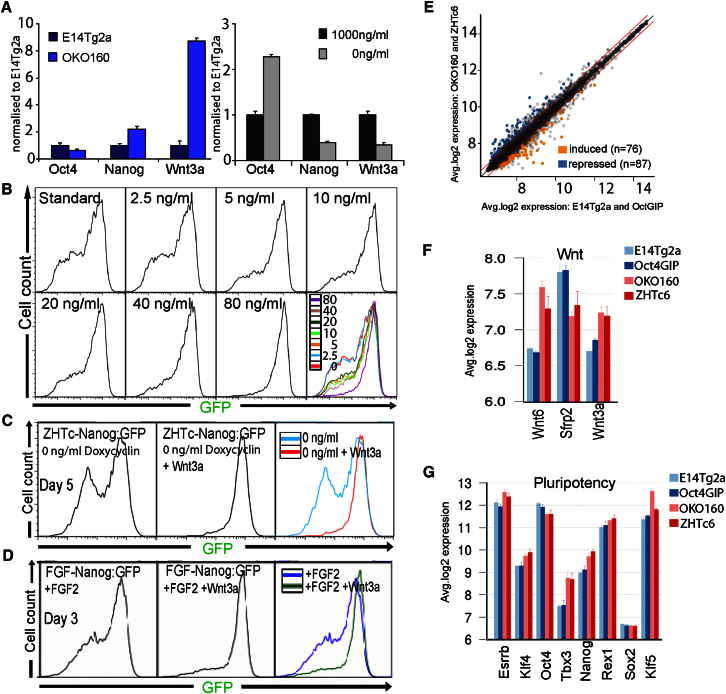
Wnt and Transcription Factor Distinctions between *Oct4*^+/+^ and *Oct4*^+/−^ ESCs (A) qPCR analysis of E14Tg2a and OKO160 ESCs for the indicated transcripts (left) and ZHTc-Nanog:GFP ESCs treated with the indicated doxycycline dose for 4 days (right). Error bars represent SD; n = 3. (B) FACS analysis of Nanog:GFP expression in Tg2a-Nanog:GFP cells treated with the indicated Wnt3a concentrations for 3 days. (C) FACS analysis of Nanog:GFP expression in ZHTc-Nanog:GFP cells treated without doxycycline in the presence or absence of Wnt3a (80 ng/ml). (D) FACS analysis of Nanog:GFP expression in FGF-Nanog:GFP cells treated with FGF2 in the presence or absence of Wnt3a (80 ng/ml). (E) A comparison of the mean-quantile-normalized gene-expression levels in *Oct4*^+/+^ (E14Tg2a and OctGIP) and *Oct4*^+/−^ (OKO160 and ZHTc6) ESCs. Genes differentially and consistently overexpressing (“induced” by Oct4) or underexpressing (“repressed” by Oct4) in *Oct4*^+/+^ versus *Oct4*^+/−^ (|log2FC| ≥ log2(1.25), FDR ≤ 0.1; see Experimental Procedures) are highlighted in blue and orange, respectively. Boundaries for a 1.25× fold change are shown in red. (F) Average quantile-normalized expression intensities for Wnt signaling and pluripotency-related (G) genes. Error bars represent SEM. See also Figure S6 and Tables S1, S2, and S3.

Previously, Wnt signaling has been reported to act synergistically with LIF in order to support ESC self-renewal (Hao et al., 2006; Ogawa et al., 2006). Interestingly, *Oct4*^+/−^ ESCs express increased Wnt3a mRNA (Figure 6A) with Oct4 induction downregulating Wnt3a (Figure 6A). Therefore, the potential of Wnt3a to regulate Nanog heterogeneity was assessed by FACS. Wnt3a blocked the emergence of a heterogeneous Nanog expression profile from Tg2a-Nanog:GFP cultures in a dose-dependent manner (Figure 6B). Wnt3a also prevented Oct4-induced Nanog heterogeneity in *Oct4*^+/−^ ESCs (Figure 6C). Moreover, the ability of FGF to induce Nanog heterogeneity in *Oct4*^+/+^ ESCs was highly attenuated by Wnt3a (Figure 6D).

To further investigate molecular distinctions between *Oct4*^+/+^ and *Oct4*^+/−^ ESCs, we performed microarray analysis on two *Oct4*^+/+^ and two *Oct4*^+/−^ ESC lines (Figures 6E and S6). This identified 163 genes that differed consistently between the two *Oct4*^+/+^ and two *Oct4*^+/−^ ESC lines (|log_2_FC| ≥ log_2_(1.25), false discovery rate [FDR] ≤ 0.1; see Experimental Procedures). Functional enrichment analysis identified the biological processes “Wnt receptor signaling pathway” (FDR = 0.0609) and “embryonic pattern specification” (FDR = 0.0397) as the only gene ontology terms enriched in the candidate list. In addition to Wnt3a, Wnt6 was increased in *Oct4*^+/−^ ESCs, whereas the Wnt antagonist Sfrp2 was decreased (Figure 6F). Furthermore, several pluripotency transcription factors were elevated in *Oct4*^+/−^ ESCs (Figure 6G).

### Oct4 and Nanog Occupancy in *Oct4*^+/−^ ESCs

**Figure 7:**
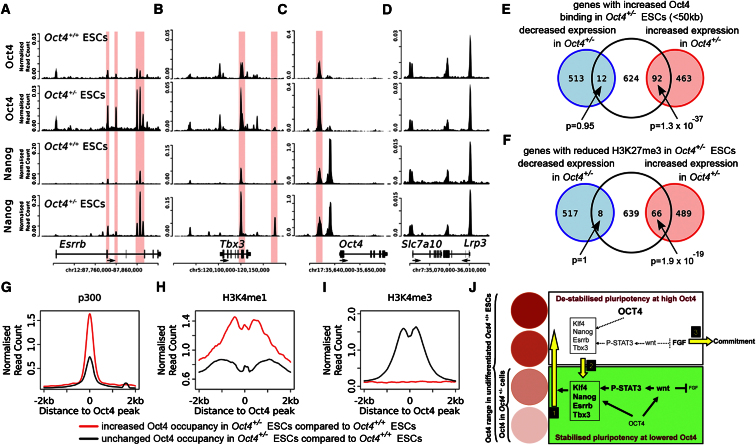
Genome-Wide Analysis for Oct4 and Nanog Occupancy in *Oct4*^+/−^ ESCs (A–D) Chromatin immunoprecipitation sequencing profiles of Oct4 and Nanog for Esrrb (A), Tbx3 (B), Oct4 (C), and Slc7a10 (D) gene loci in *Oct4*^+/+^ ESCs and *Oct4*^+/−^ ESCs . Read counts are normalized by the number of uniquely mapped reads. (E) Genes with increased Oct4 binding within 50 kb of the transcription start site (TSS) in *Oct4*^+/−^ ESCs are enriched in the gene set showing increased expression in *Oct4*^+/−^ ESCs; significance was estimated by Fisher’s exact test. (F) Genes with reduced levels of H3K27me3 in *Oct4*^+/−^ ESCs are enriched in the gene set showing increased expression in *Oct4*^+/−^ ESCs; significance was estimated by Fisher’s exact test. (G–I) Average read count for p300 (G), H3K4me1 (H), and H3K4me3 (I) at Oct4 binding sites shared between *Oct4*^+/+^ and *Oct4*^+/−^ ESCs (black) or showing increased Oct4 occupancy in *Oct4*^+/−^ ESCs (red). (J) A model of the pluripotency GRN. ESCs express a range of Oct4 concentrations (red circles), only the lower of which are present in *Oct4*^+/−^ ESCs (green box). In this lower state, Oct4 binds strongly to key regulatory genes, encoding heterogeneously expressed transcription factors and Wnt signaling components. The increased Wnt signaling results in enhanced phospho-STAT3 levels in response to LIF, further reinforcing the expression of heterogeneously expressed pluripotency transcription factors. This produces upward pressure on the *Oct4* gene, driving cells into the high Oct4 concentration range (yellow arrow 1). At these high Oct4 concentrations (white box), the Wnt system is not activated and Oct4 binding to transcription factor loci is weakened. Exit of cells from this state can result either from the residual GRN activity, causing downward fluctuation in the Oct4 expression (yellow arrow 2) or from the cell experiencing an effective commitment signal (yellow arrow 3). See also Figure S7 and Tables S1, S3, and S4.

Next, we investigated the genome-wide binding profiles of Oct4 and Nanog in both *Oct4*^+/−^ and *Oct4*^+/+^ ESCs. For both Oct4 and Nanog, the majority of loci are shared between *Oct4*^+/+^ and *Oct4*^+/−^ ESCs (Figures S7A and S7B). However, striking differences in the binding intensity exist between *Oct4*^+/+^ and *Oct4*^+/−^ ESCs. At genes encoding pluripotency regulators (Esrrb, Tbx3, Oct4, Klf5, and Wnt6), specific loci with increased Oct4 and/or Nanog occupancy were identified (Figure 7A, 7B, 7C, S7C, S7D, and S7E). Such an increase was not found at other loci (e.g., Figure 7D). Next, we asked whether genes with increased Oct4 occupancy are associated with gene expression. Genes near Oct4-binding events that are enhanced in *Oct4*^+/−^ ESCs are significantly enriched in the set of genes with higher expression in *Oct4*^+/−^ ESCs (p = 1.7 × 10^−37^; Figure 7E), whereas Oct4-binding events reduced in *Oct4*^+/−^ ESCs occur near genes with higher expression in *Oct4*^+/+^ ESCs (p = 1.2 × 10^−8^, Figure S7F). This change in gene expression is reflected in epigenetic differences. Genes where the promoter shows reduced levels of the repressive histone modification H3K27me3 in *Oct4*^+/−^ ESCs in comparison to *Oct4*^+/+^ ESCs show higher expression in *Oct4*^+/−^ ESCs (Figure 7F; p = 1.9 × 10^−19^). Altogether, this indicates that qualitative differences of Oct4 occupancy along with epigenetic differences are involved in regulating the robustness of the pluripotent state via effects on the pluripotency GRN.

Surprisingly, even though Oct4 protein is reduced in *Oct4*^+/−^ ESCs, the binding sites with the strongest difference between *Oct4*^+/+^ and *Oct4*^+/−^ ESCs show increased occupancy in *Oct4*^+/−^ ESCs. These *Oct4*^+/−^-specific Oct4 binding sites occur largely at genomic loci associated with p300 and H3K4me1, but not H3K4me3 (Figures 7G, 7H, and 7I). p300 and H3K4me1 are classical marks for enhancer regulatory sites, whereas H3K4me3 is highly abundant at proximal promoters. In addition, the sites that show enhanced Oct4 occupancy in the *Oct4*^+/−^ ESCs are generally further from the transcription start sites (Figure S7G). Moreover, loci showing enhanced occupancy by Oct4 also exhibit enhanced co-occupancy by Nanog (Figure S7H).

These data point toward an increase in Oct4 occupancy at enhancer regulatory regions in the *Oct4*^+/−^ ESCs. The proximal and distal enhancers of *Oct4* (Figure 7C) are of particular interest. The distal enhancer, which is associated with preimplantation embryos and ESCs (Yeom et al., 1996; Han et al., 2010), shows increased binding of Oct4 and Nanog in *Oct4*^+/−^ ESCs. The proximal enhancer, which is associated with postimplantation epiblast and EpiSCs (Yeom et al., 1996), shows decreased binding of Nanog. Thus, reduced Oct4 protein levels in *Oct4*^+/−^ ESCs appear to induce a shift in Oct4 binding preferences toward a higher occupancy of key regulatory elements, thereby strengthening the regulatory network that maintains robust pluripotency.

## Discussion

Although the heterogeneous expression of pluripotency transcription factors has the capacity to generate multiple outcomes from a single functionally defined cell type (Canham et al., 2010; Chambers et al., 2007; Glauche et al., 2010; Kalmar et al., 2009; Singh et al., 2007), the factors controlling heterogeneity are only beginning to be elucidated. Nanog autorepression has recently been demonstrated (Fidalgo et al., 2012; Navarro et al., 2012) with this contributing to Nanog heterogeneity (Navarro et al., 2012). In addition, Satb1, Satb2, and Ezh2 have been reported to influence Nanog heterogeneity (Savarese et al., 2009; Villasante et al., 2011). In this study, we identify an origin for transcription factor heterogeneity within the circuitry of the pluripotency GRN. Multiple *Oct4*^+/−^ ESC lines have a unimodal high Nanog expression profile and lack Nanog-low undifferentiated cells. Heterogeneity is caused by a higher range of Oct4 levels present in WT ESC cultures because increasing the Oct4 concentration range toward the WT distribution switches Nanog to a bimodal heterogeneous profile.

The fact that *Oct4*^+/−^ cultures not only lack cells expressing the highest Oct4 concentrations present in *Oct4*^+/+^ cultures but are also essentially devoid of the differentiated Oct4-negative cells that are readily detectable in WT cultures is of no small interest here. These findings have two important implications. First, the full range of Oct4 concentrations present in the *Oct4*^+/−^ population is within the distribution of expression levels observed both in WT ESC cultures and in the WT ICM, indicating that the Oct4 protein levels in *Oct4*^+/−^ cells are physiologically relevant. Second, the appearance of differentiated Oct4-negative cells in ESC cultures is part of the normal network configuration assembled by WT Oct4 levels and does not require excessive Oct4 expression.

The more homogeneous expression of transcription factors present in *Oct4*^+/−^ ESCs results in delayed differentiation in comparison to WT ESCs. Importantly, restoring Nanog heterogeneity in *Oct4*^+/−^ ESCs by transiently increasing the Oct4 level re-established normal differentiation. Furthermore, enforced expression of Nanog prevents differentiation induced by Oct4 elevation. Altogether these findings offer an explanation for the original observation that an increase in Oct4 can cause differentiation (Niwa et al., 2000). Rather than being a consequence of Oct4 action at elevated concentrations, our results show that Oct4 acts at physiological levels to provide a population of Nanog-low cells responsive to differentiation cues.

Our results establish unequivocally that a state of homogeneous transcription factor expression and robust self-renewal can exist without the need for pharmacological signaling pathway inhibition (Ying et al., 2008). The reasons for this are manifold. First, *Oct4*^+/−^ ESCs exhibit an increased sensitivity to LIF. This can be seen by enhanced STAT3 phosphorylation, enhanced colony-forming capacity at low LIF concentrations, and enhanced maintenance of unimodally high Nanog:GFP expression at low LIF concentrations. This increased sensitivity to LIF is accompanied by a resistance to the induction of Nanog heterogeneity and differentiation in response to FGF. Interestingly, *Oct4*^+/−^ ESCs respond to FGF by increasing ERK phosphorylation, suggesting that the lack of a full response to FGF in these cells is due to either the interception and elimination of effective signaling downstream of ERK or the dominance of an opposing signal. FGF4 mRNA expression is elevated in *Oct4*^+/−^ cells, which may reflect dysregulation of the FGF signaling system in these cells. The combined inhibition of ERK and GSK3b has been spectacularly successful for the derivation of ESCs from recalcitrant strains of mice (Ying et al., 2008) and from other species (Buehr et al., 2008; Li et al., 2008). Perhaps the inability to derive ESCs from the majority of non-129 mouse strains is due to the expression level of Oct4 being beyond the range that can be stabilized in culture by LIF.

*Oct4*^+/−^ cells also exhibit elevated expression of Wnt6 and Wnt3a and decreased expression of Sfrp2, which is indicative of a shift in the Wnt signaling system. Wnt3a has previously been reported to act in synergy with LIF on ESCs (Ogawa et al., 2006); although, without LIF, Wnt3a cannot maintain an undifferentiated ESC state. Indeed, in the presence of Wnt3a, LIF concentrations as low as 6 U/ml can support undifferentiated colony formation by WT ESCs (Ogawa et al., 2006). Here, we show that, at this LIF concentration, the majority of *Oct4*^+/−^ ESCs colonies are completely undifferentiated. Supplementation of ESC cultures with Wnt3a has also been reported to block the transition from ESCs to EpiSCs but, again, only when LIF is also added (ten Berge et al., 2011). Not only do we observe a retarded transition to EpiSCs by *Oct4*^+/−^ ESCs, but we also find that *Oct4*^+/−^ ESCs can be clonally propagated in LIF-N2B27 in the absence of either BMP or signaling inhibitors (Ying et al., 2008).

Although the differentiation of *Oct4*^+/−^ ESCs to an EpiSC state is delayed, *Oct4*^+/−^ EpiSCs can be established. An interesting property of *Oct4*^+/−^ EpiSCs is the capacity to form undifferentiated ESC-like colonies when replated in LIF-fetal calf serum (FCS). It has been reported previously that EpiSCs can revert to an ESC-like state after culture on feeder cells (Bao et al., 2009). However, this reversion is slow, requiring >20 days. In contrast, *Oct4*^+/−^ EpiSCs form ESC-like colonies in the absence of feeder cells within 6 days. This suggests that the distinction between the ESC and EpiSC pluripotent states is less clear-cut in cell populations expressing a reduced Oct4 concentration range. This may indicate that the higher Oct4 concentrations present in WT ESCs are important in imparting unidirectionality upon developmental progression.

It is of interest that *Oct4*^+/−^ ESCs show increased STAT3 phosphorylation in response to LIF. This may be related to the altered expression of Wnt signaling ligands in *Oct4*^+/−^ ESCs, given that evidence has been presented that Wnt3a may increase STAT3 expression (Hao et al., 2006). Although Oct4 upregulation and a decrease in LIF concentration result in a similar differentiation phenotype (Niwa et al., 2000), no connection has previously been made between Oct4 and STAT3 signaling. Here, we show that the degree of STAT3 phosphorylation varies with levels of Oct4 and surprisingly, in *Oct4*^+/−^ ESCs, phosphorylated STAT3 can be detected in almost every cell. This contrasts with WT ESCs and suggests that insufficient or attenuated STAT3 signaling may contribute to ESC heterogeneity. These results suggest that *Oct4*^+/−^ ESCs are primed to respond to LIF via enhanced STAT3 phosphorylation. The possibility that enhanced STAT3 signaling may have a dominant effect over signals antagonistic to ESC self-renewal such as ERK has been suggested (van Oosten et al., 2012). Therefore, our findings that *Oct4*^+/−^ EpiSCs have a higher reprogramming efficiency than WT EpiSCs may, at least in part, be due to the enhanced phosphorylation of STAT3 in *Oct4*^+/−^ cells.

LIF has been reported to be upstream of transcription factor expression (Niwa et al., 2009). Therefore, we propose that enhanced STAT3 phosphorylation leads to the more homogenous expression of pluripotency factors such as Nanog, Klf4, and Esrrb. Under such conditions, the profile of Oct4 binding shifts to sites more distal to transcription start sites. A prime example is the *Oct4* gene. The distal *Oct4* enhancer, which is more functional than the proximal enhancer in ESCs and the ICM than in EpiSCs and the postimplantation epiblast (Han et al., 2010; Yeom et al., 1996), is more fully occupied by Oct4 in *Oct4*^+/−^ ESCs where the Oct4 concentration is reduced. These observations suggest that the robust network configuration of *Oct4*^+/−^ ESCs reflects an early developmental state.

Theoretical models have been proposed to explain heterogeneous Nanog expression (Glauche et al., 2010; Kalmar et al., 2009). Both involve negative feedback on *Nanog*, and one model proposes Oct4 as a negative regulator of *Nanog* (Kalmar et al., 2009). Our work establishes the veracity of this conjecture but reveals that a state of nondynamic unimodal Nanog expression exists at diminished Oct4 levels.

Our findings can be incorporated into a model in which Oct4 plays two diverse roles (Figure 7J). At the lower range of Oct4 concentrations found in WT ESCs and typified by *Oct4*^+/−^ ESCs, Oct4 binds strongly to chromatin at key regulatory elements, directing the homogenous expression of pluripotency transcription factors and the expression of Wnt signaling components. This increased Wnt signaling activity enhances cell sensitivity to LIF (Hao et al., 2006; Ogawa et al., 2006), further re-enforcing the pluripotency GRN (Niwa et al., 2009) and making cells more refractory to differentiation signals such as FGF. This state can be maintained in *Oct4*^+/−^ ESCs because the upward pressure of the re-enforced pluripotency transcription factors on the *Oct4* loci cannot enforce Oct4 protein elevation because of the deletion of one of the *Oct4* alleles. However, in *Oct4*^+/+^ cells, this movement into the higher Oct4 concentration range can occur. Then, the Wnt signaling system is no longer stimulated by Oct4, and the pluripotency transcription factors are expressed at a lower level. ESCs may exit this Oct4-high state by diminishing Oct4 expression as a consequence of the weakened transcription factor network strength, thereby reverting to an Oct4-low concentration range or by responding to a commitment signal (e.g., FGF) and differentiating.

A priori, one might have expected the pluripotency GRN to have been optimally configured to support robust ESC self-renewal. In this respect, the demonstration that such robust ESC self-renewal is anchored through a network configuration orchestrated by reduced Oct4 concentrations is highly unexpected. This indicates that the control of pluripotent cell identity is naturally arranged in ESCs and ICM cells to provide heterogeneity. Consequently, stem cell self-renewal and rapid responsiveness to differentiation cues can simultaneously reside in a single stem cell population in which individual cells experience the same environmental stimuli. When one considers the situation in the embryo, it is perhaps less surprising that the network is not optimized for extended self-renewal, given that progression beyond the pluripotent state is required for orderly development. It will be of considerable interest to determine whether similar strategies are used in other stem cell systems to likewise facilitate rapid responses to changing physiological environments.

## Experimental Procedures

### ESC Culture

ESCs were cultured in GMEMβ with 10% FCS and 100 U/ml LIF on gelatinized tissue culture flasks at a density of 2.5–10 × 10^4^/cm^2^ (Smith, 1991). Differentiation was induced by LIF withdrawal (Smith, 1991) with a neural differentiation protocol (Ying et al., 2008) or into EpiSCs, as described previously (Osorno et al., 2012). Recombinant Wnt3a (R&D Systems) and recombinant Fgf2 (R&D) were used as indicated. JAK inhibitor I (Calbiochem) was used at 0.6 mM and PD0325901 (Axon Medchem) and Chiron 99021 (Axon) were used as described previously (Ying et al., 2008). Introduction of the Nanog:GFP reporter by homologous recombination was performed and verified as described previously (Chambers et al., 2007).

### Colony-Forming Assay

The colony-forming assay was performed by seeding 600 cells in a well of a six-well plate (60 cells/cm^2^). Cells were plated in GMEMβ-FCS with or without LIF or in N2B27 (Ying et al., 2008) supplemented with recombinant LIF (Sigma-Aldrich) and/or recombinant BMP4 (R&D) as indicated and cultured in a 37°C, 7% CO_2_ incubator. After 7 days, cell staining was performed with an Alkaline Phosphatase Detection Kit (Sigma-Aldrich).

### Immunofluorescence Quantifications

For ESC quantifications, cells were segmented on the basis of DAPI with the nuclei detection algorithm of PerkinElmer’s Acapella Image Analysis Suite. For each nucleus, mean fluorescence for Oct4 and Nanog were computed.

For embryo quantification, the fluorescence signal in each nucleus of whole-mount immunostained blastocysts were quantified with an automated pipeline as described previously (Osorno et al., 2012), except that preprocessed DAPI-stained nuclei of RGB stack images were segmented with the FARSIGHT Toolkit (Al-Kofahi et al. 2010) (http://www.farsight-toolkit.org/) and incorrectly segmented nuclei were manually rectified with the FARSIGHT Nucleus Editor.

## Supplemental Information

Document S1. Supplemental Experimental Procedures, Figures S1–S7, and Tables S1 and S2

Table S3. A Spreadsheet of an Integrated Analysis of Gene Expression Data, Related to Figure 6

Table S4. Spreadsheet of Oct4 and Nanog Chromatin Binding Data, Related to Figure 7
