# Supplementary material for: Reduced Oct4 Expression Directs a Robust Pluripotent State with Distinct Signaling Activity and Increased Enhancer Occupancy by Oct4 and Nanog
Source: Cell Stem Cell. 2013 May 2;12(5):531–45. doi: 10.1016/j.stem.2013.04.023 (PMC3650585; doi:10.1016/j.stem.2013.04.023)
Supplement: Document S1. Supplemental Experimental Procedures, Figures S1–S7, and Tables S1 and S2 [file mmc1.pdf]

Cell Stem Cell, Volume *12*
Supplemental Information

Reduced Oct4 Expression Directs a Robust Pluripotent State with Distinct Signaling Activity and Increased Enhancer Occupancy by Oct4 and Nanog

Violetta A. Karwacki-Neisius, Jonathan Göke, Rodrigo Osorno, Florian Halbritter, Jia Hui Ng, Andrea Y. Weiße, Frederick C. K. Wong, Alessia Gagliardi, Nicholas P. Mullin, Nicola Festuccia, Douglas Colby, Simon R. Tomlinson, Huck-Hui Ng, and Ian Chambers

Figures:

Figure S1 with legend, related to Figure 1.

Figure S2 with legend, related to Figure 2.

Figure S3 with legend, related to Figure 3.

Figure S4 with legend, related to Figure 3.

Figure S5 with legend, related to Figure 5.

Figure S6 with legend, related to Figure 6.

Figure S7 with legend, related to Figure 7.

Tables:

Table S1, related to Figures 1–7.

Table S2, related to Figures 1, 3, and 6.

Supplemental Experimental Procedures

Supplemental References

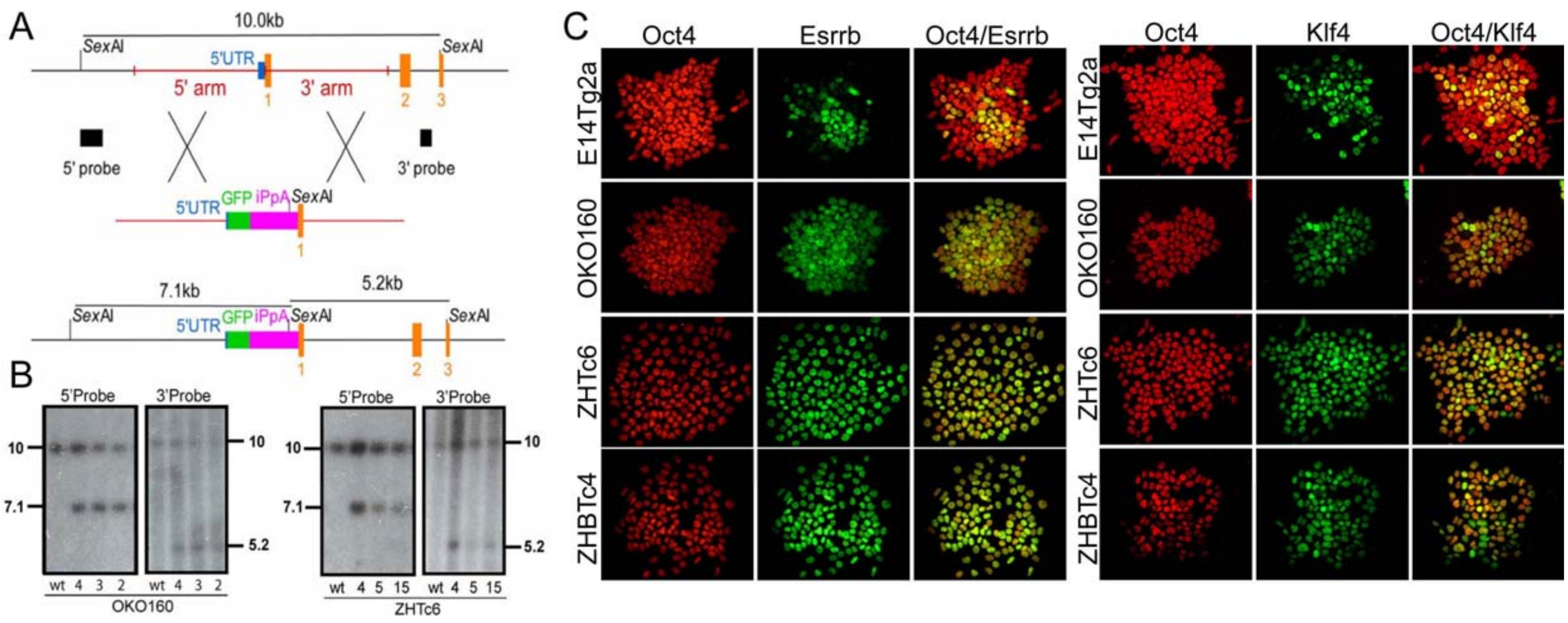

Figure S1. Genetic Construction of Nanog:GFP Reporter Derivatives of Cells Expressing Reduced Oct4 Levels, Related to Figure 1

(A) The 5' end of the *Nanog* gene is shown schematically at the top. Exons, orange; 5'UTR, blue; homology arms used for construction of the targeting vector, red. eGFP was inserted between the homology arms precisely at the Nanog AUG codon in the targeting construct shown in the middle. GFP expression is linked through an IRES to puromycin resistance encoded by the *pac* gene and followed by a polyA site (iPpA).
(B) Southern blot analysis of *Sex*AI digested genomic DNA from targeted clones from OKO160 (left) and ZHTc6 (right) cells. Southern shows one wild type clone with a 10 kb band only and three independent correctly targeted clones.
(C) Immunoluorescence analysis in Oct4$^{+/+}$ (E14Tg2a) and Oct4$^{+/-}$ (OKO160, ZHTc6 and ZHBTc4) ES cells for Esrrb (left) and Klf4 (right).

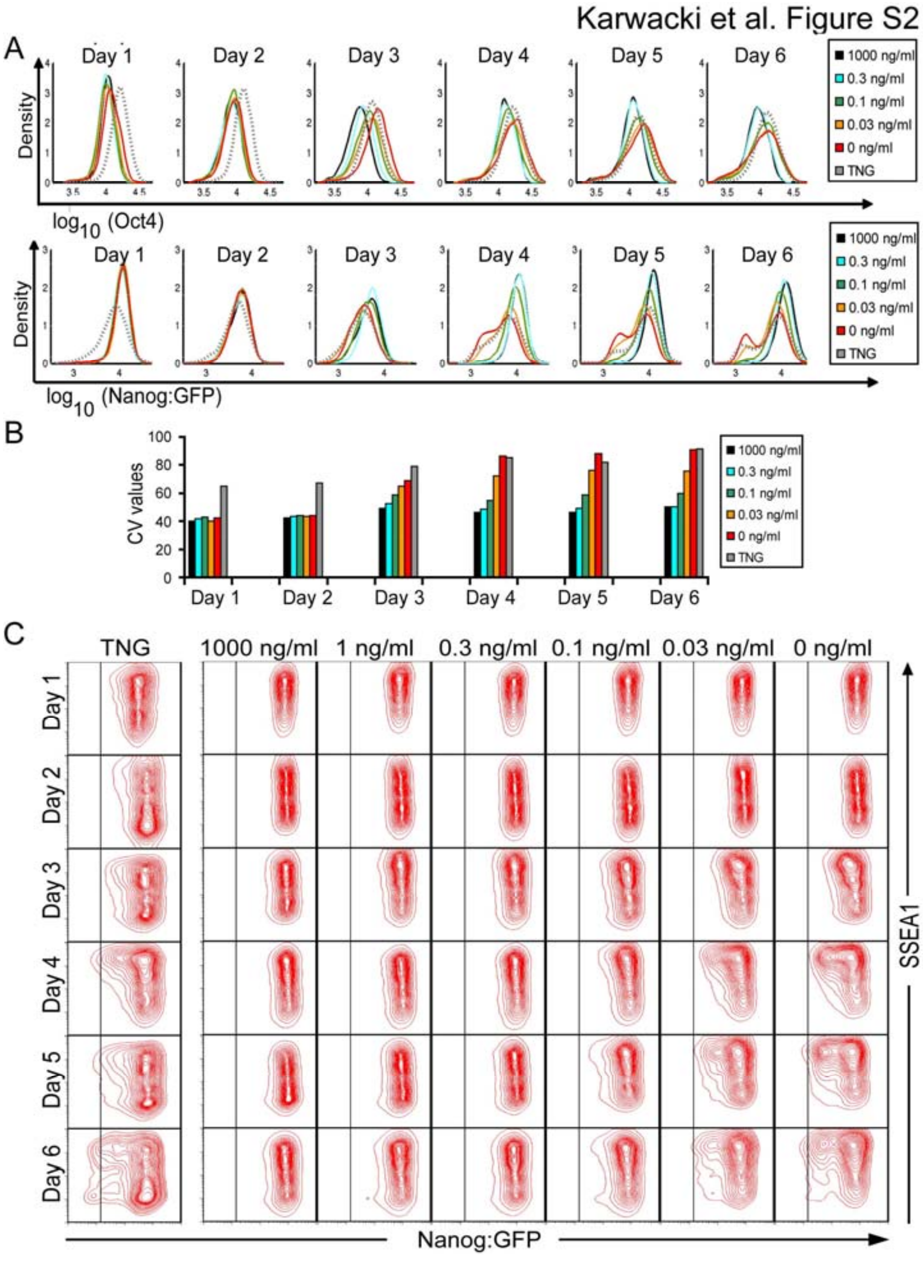

Figure S2. Titrated Elevation of Oct4 Levels Restores Nanog Heterogeneity, Related to Figure 2

(A) Intracellular FACS staining for Oct4 and analysis for Nanog:GFP expression in ZHTc-Nanog:GFP cells treated with the indicated dose of doxycycline (right) over the time course of six days. Tg2a-Nanog:GFP (TNG) preselected in puromycin to remove Nanog:GFP-negative cells were analysed in parallel after removal of puromycin at day 0.

(B) Coefficient of variation for the Nanog:GFP distribution data shown in (A).

(C) FACS analysis for Nanog:GFP (x-axis) and SSEA1 (y-axis) expression in ZHTc-Nanog:GFP cells treated with the indicated doxycycline concentrations (top) for the indicated times (left margin). Tg2a-Nanog:GFP (TNG) cells were analysed in parallel.

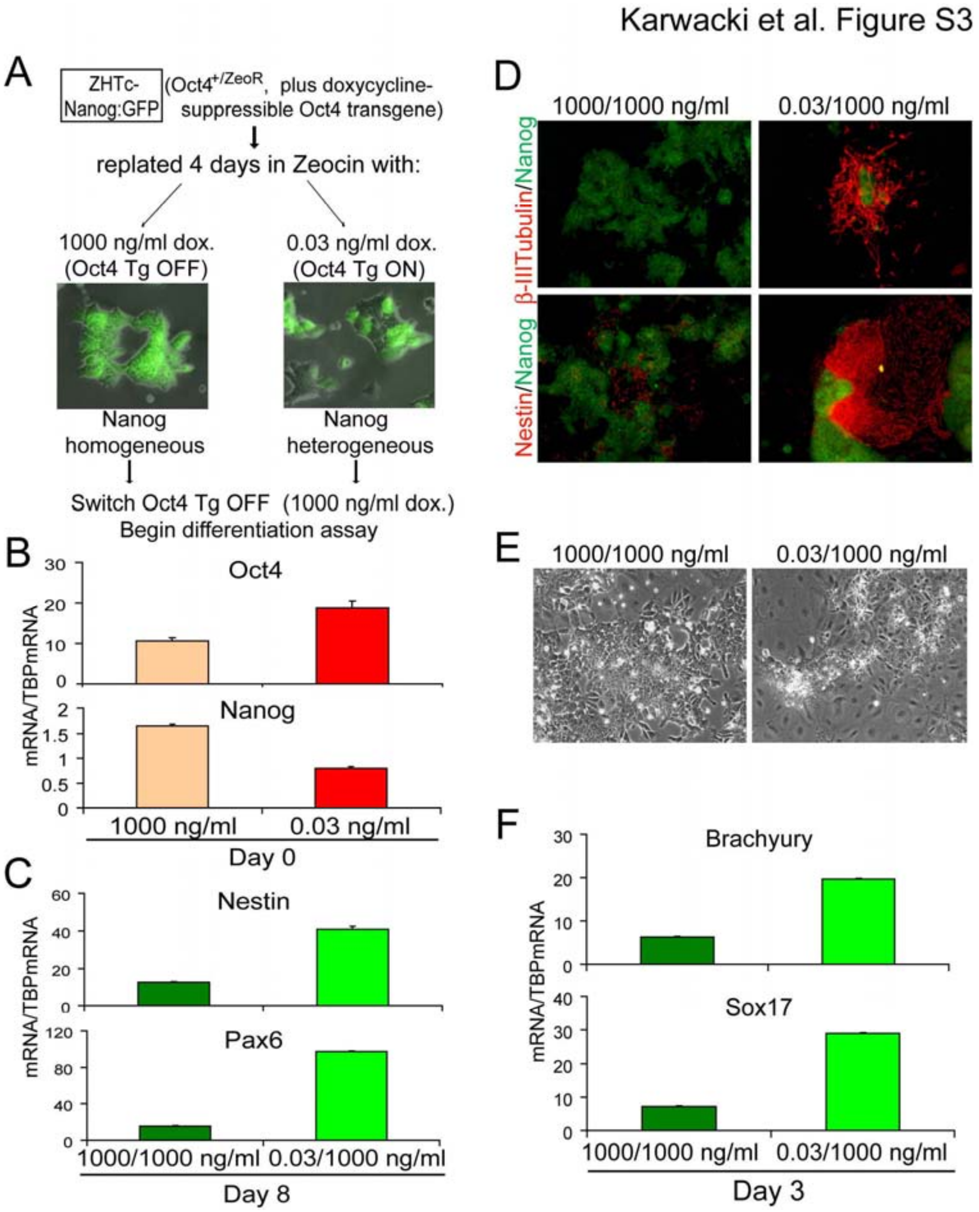

Figure S3, Restoration of Nanog Heterogeneity in *Oct4*$^{+/-}$ Cells Rescues Retarded Differentiation, Related to Figure 3

(A) Experimental scheme. ZHTc-Nanog:GFP cells were treated with the indicated dose of doxycycline before and during differentiation.
(B) qPCR analysis of mRNA expression in ZHTc-Nanog:GFP cells (day 0). Doxycycline treatment is indicated. Errors bars: standard deviation (n=3).
(C) qPCR analysis of mRNA expression. Errors bars: standard deviation (n=3) and (D) immunofluorescence analysis at day 8 of neural differentiation.
(C–F) The doxycycline concentrations applied before (first dose) and during induction (second dose) of differentiation are indicated. (E) cell morphology at day 3 of LIF withdrawal. (F) Q-PCR analysis at day 3 of LIF withdrawal. Errors bars: standard deviation (n=3).

Karwacki et al. Figure S4

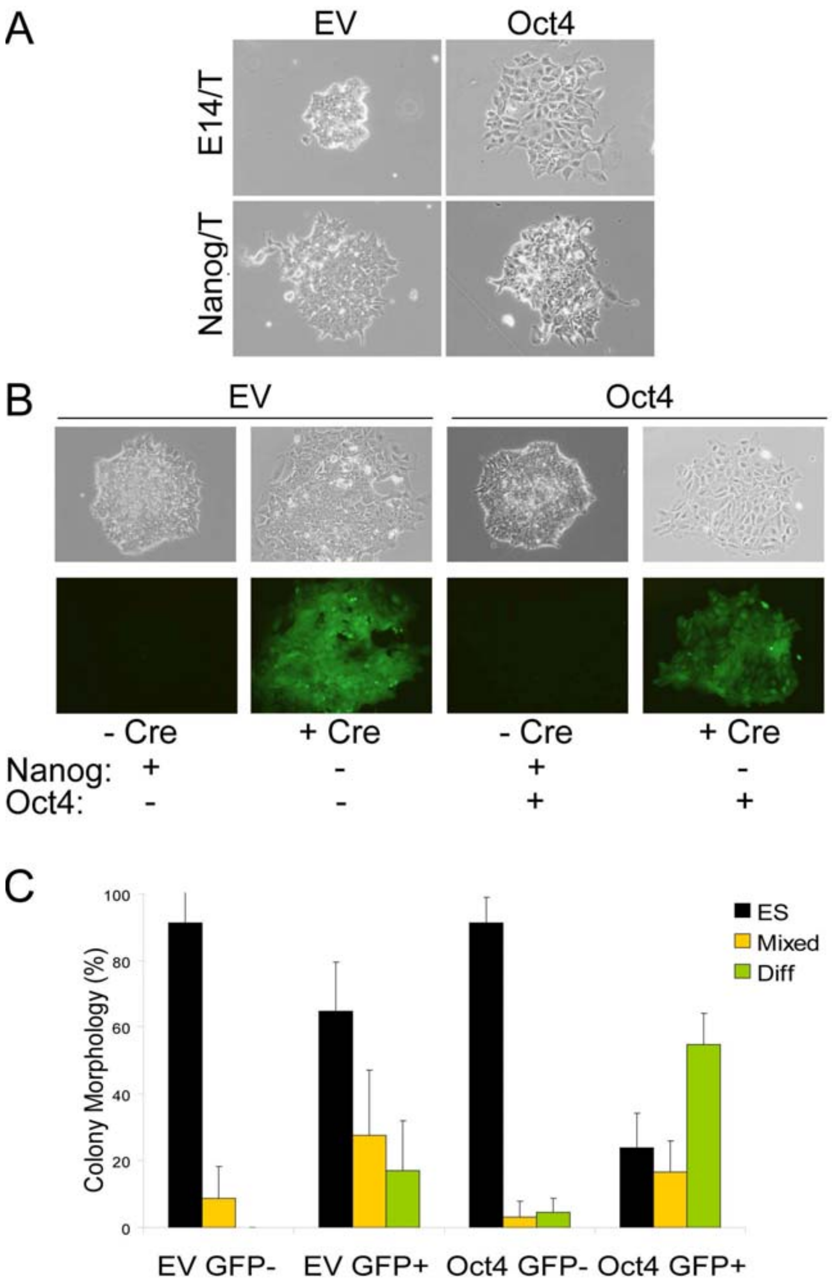

Figure S4, Functional Influences of Nanog Heterogeneity, Related to Figure 3

(A) Morphology of control cells (E14/T) and cells carrying a loxP-flanked Nanog transgene (Nanog/T) following Oct4 episomal supertransfection.
(B and C) Representative morphologies and (C) colony quantitation following Cre excision of Nanog (resulting in constitutive expression of GFP) from Nanog/T-empty vector (EV) or Oct4 transfectants. Errors bars: standard deviation of 3 biological replicates.

Karwacki et al. Figure S5

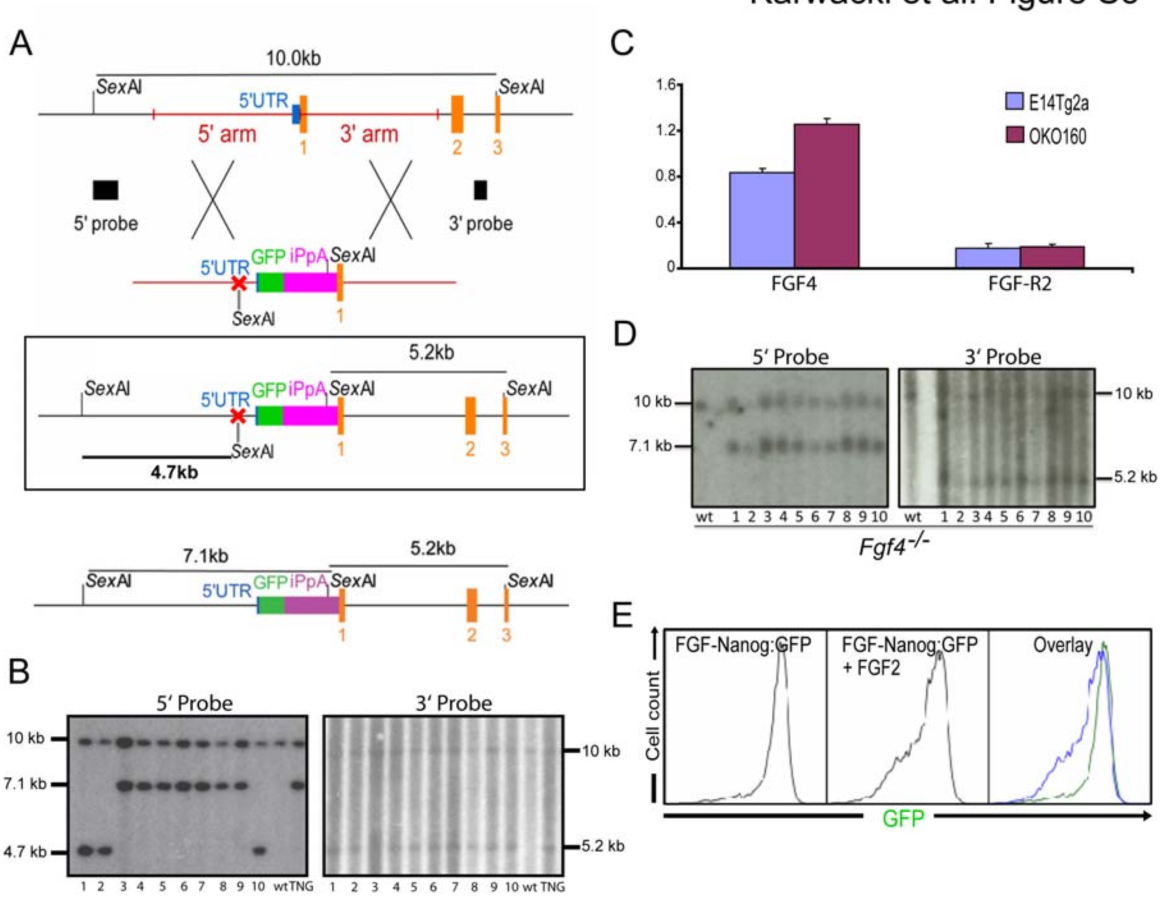

Figure S5, Signalling Influences, Related to Figure 5

(A) The Oct4 binding site was mutated into a *Sex*A1 restriction site (red cross) in the targeting vector. This mutated Oct4 binding site lies 3250bp from the 5′end and 370bp from the 3′end of the 5′ homology arm. The positions of the flanking probes used for Southern analysis and the fragment sizes produced by *Sex*AI digestion are indicated. Black box indicates the mutated GFP reporter allele. The non mutated allele is shown at the bottom of (A).

(B) Southern blot analysis of *Sex*AI digested genomic DNA from E14Tg2a cells in which the Oct4 mutant targeting vector was introduced by homologous recombination. The loss of the Oct4 binding sequence is visible in the 5′ Blot in the appearance of a 4.7 kb band. The 3′ Blot should show for mutated and non-mutated cells a 10 and 5.2 kb band. Wild type cells show one 10kb band only.

(C) qPCR analysis on the indicated cell lines for FGF4 and FGF-Receptor2 (FGF-R2). Errors bars: standard deviation (n=3)

(D) Blot analysis of the genetic construction of *Nanog:GFP* reporter derivatives of $FGF4^{-/-}$ ES cells as described in Supplementary Figure 1.

(E) $FGF4^{-/-}$ ES cells correctly targeted on the Nanog locus (FGF-Nanog:GFP) treated and untreated with FGF2 over the time course of 2 days.

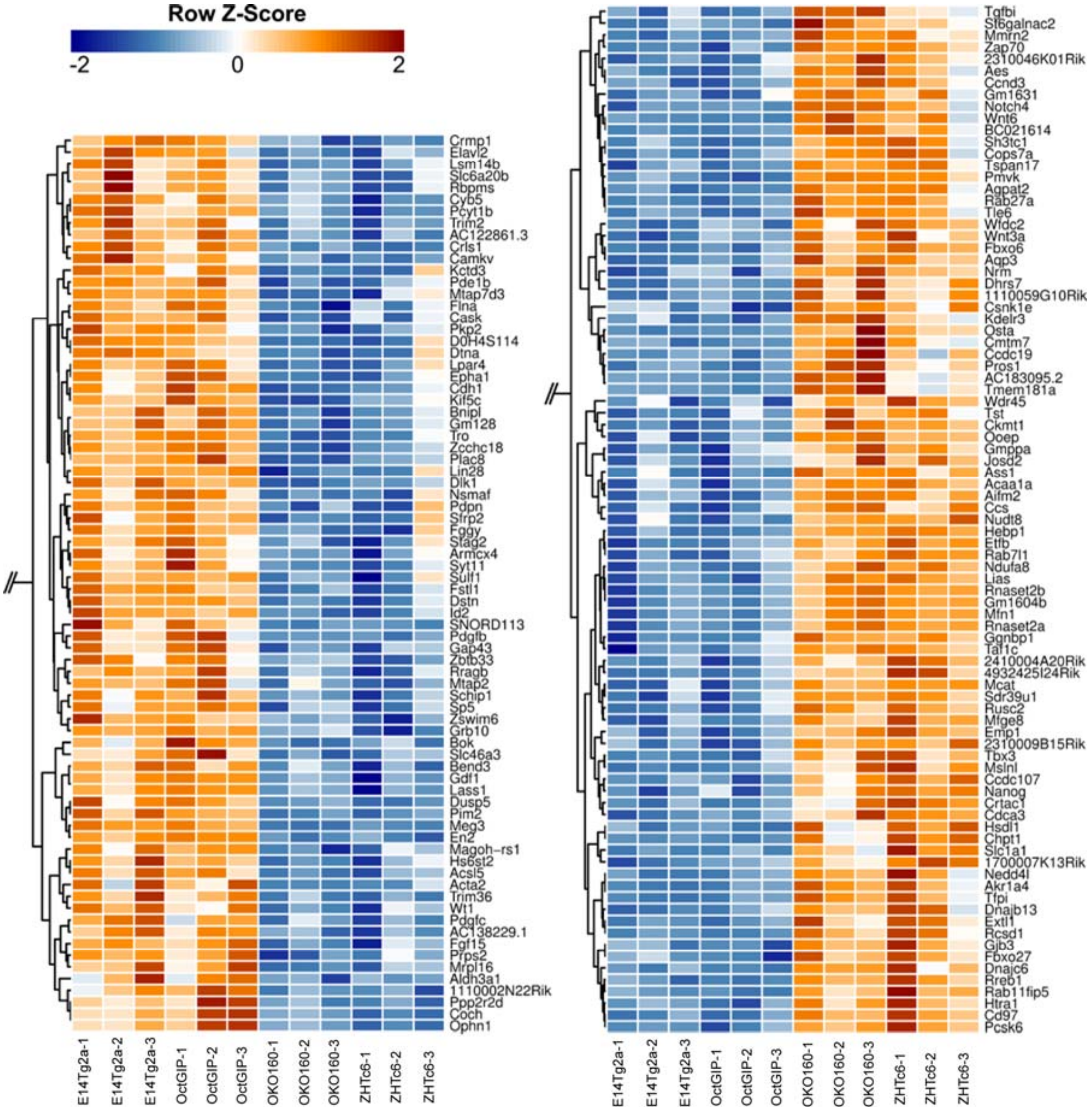

Figure S6, Gene Expression Heatmap, Related to Figure 6

Heatmap representation of quantile-normalised gene expression intensities in *Oct4*$^{+/+}$ (E14Tg2a, OctGIP) and *Oct4*$^{+/-}$ (OKO160 and ZHTc6) mouse ESCs. Rows (genes) have been reordered by hierarchical clustering with complete linkage using correlation dissimilarity as distance. Colours have been scaled by row (gene) to highlight relative changes between genes in the different cell lines. Shown are all genes that were consistently differentially expressed in all cell line comparisons ($|\log2FC| >= \log2(1.25)$, FDR <= 0.1; see Methods). The heatmap has been split into the two major clusters for representation purposes.

Karwacki et al. Figure S7

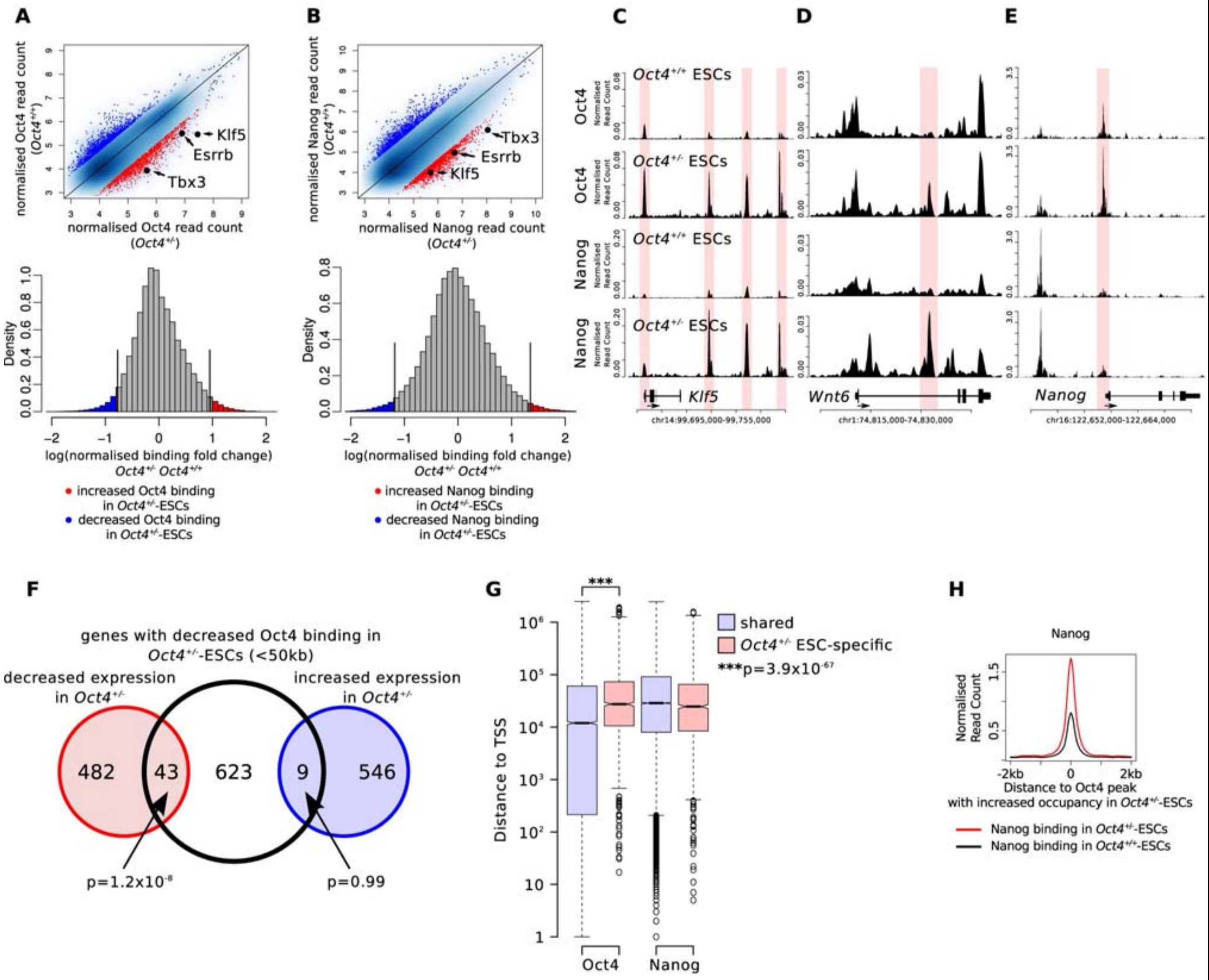

Figure S7, Related to Figure 7

(A) (top) Scatterplot showing the normalised number of read counts at Oct4 binding sites in *Oct4*$^{+/+}$ and *Oct4*$^{+/-}$ ESCs. (bottom) Histogram of normalised binding intensity fold changes for Oct4 in *Oct4*$^{+/-}$ and *Oct4*$^{+/+}$ ESCs. The top 1500 binding sites that show the strongest increase in binding in *Oct4*$^{+/-}$ are marked in red, the top 1500 binding sites that show the strongest decrease in binding are marked in blue.

(B) (top) Scatterplot showing the normalised number of read counts at Nanog binding sites in *Oct4*$^{+/+}$ and *Oct4*$^{+/-}$ ESCs. (bottom) Histogram of normalised binding intensity fold changes for Nanog in *Oct4*$^{+/-}$ and *Oct4*$^{+/+}$ ESCs. The top 1500 binding sites that show the strongest increase in binding in *Oct4*$^{+/-}$ are marked in red, the top 1500 binding sites that show the strongest decrease in binding are marked in blue.

(C–E) The ChIP-Seq profiles of Oct4 and Nanog for Klf5 (C), Wnt6 (D) and Nanog (E) gene loci in *Oct4*$^{+/+}$ ESCs and *Oct4*$^{+/-}$ ESCs.

(F) Genes with *Oct4*$^{+/-}$ -decreased Oct4 binding within 50 kb of the transcription start site (TSS) are enriched in the gene set showing decreased expression in *Oct4*$^{+/-}$ ESCs; significance was estimated by Fisher's exact test.

(G), Distance to the nearest TSS for Oct4 and Nanog binding events which show similar binding levels between *Oct4*$^{+/+}$ and *Oct4*$^{+/-}$ ESCs (blue) and binding events that show increased Oct4 or Nanog occupancy in *Oct4*$^{+/-}$ ESCs (red). The Box shows quartiles, the black line indicates the median and the dotted line extends to the most extreme data point that is within 1.5 times the length of the box away from the box. Circles show data points beyond these boundaries. Significance was estimated by the Wilcoxon rank-sum test.

(H), Average read count for Nanog in *Oct4*$^{+/-}$ ESCs (red) and *Oct4*$^{+/+}$ ESCs (black) at Oct4 binding sites which show increased Oct4 occupancy in *Oct4*$^{+/-}$ ESCs.

Table S1. Cell Line Description, Related to Figures 1–7

CELL LINE	OCT4 GENOTYPE	GENETIC MODIFICATION
**Oct4GIP**[1]	*Oct4*$^{+/+}$	Express a randomly integrated transgene in which GFP is controlled by Oct4 regulatory elements.
**ZIN40**	*Oct4*$^{+/+}$	Express a randomly integrated lacZ-ires-neo transgene.
**OKO160**[2]	*Oct4*$^{+/-}$	An IRES-βgeopA cassette has been introduced into one Oct4 allele by homologous recombination.
**ZHTc6**[3]	*Oct4*$^{+/-}$	Contain a tTA transgene linked to Hygromycin-resistance and a Doxycycline suppressible Oct4 transgene. One Oct4 allele has been inactivated by targeting an ires-Zeo cassette.
**ZHBTc4**[3]	*Oct4*$^{-/-}$	As ZHTc6 cells but the second Oct4 allele has been inactivated by targeting an ires-Bsd cassette.
**Tg2a-Nanog:GFP**[4]	*Oct4*$^{+/+}$	An eGFP-ires-pacpA cassette inserted at the AUG codon of *Nanog*.
**OKO-Nanog:GFP**	*Oct4*$^{+/-}$	On a OKO160 background, an eGFP-ires-pacpA cassette inserted at the AUG codon of *Nanog*.
**ZHTc-Nanog:GFP**	*Oct4*$^{+/-}$	On a ZHTc6 background, an eGFP-ires-pacpA cassette inserted at the AUG codon of *Nanog*.
**AGFP7**[5]	*Oct4*$^{+/+}$	Express a randomly intergrated aGFP transgene.
**AGFP7OKO**	*Oct4*$^{+/-}$	On a AGFP7 background, an IRES-βgeopA cassette has been introduced into one Oct4 allele by homologous recombination.
**FNG-Nanog:GFP**	*Oct4*$^{+/+}$	An eGFP-ires-pacpA cassette inserted at the AUG codon of *Nanog* into *Fgf4*$^{-/-}$[6] ES cells.
**mTg2a-Nanog:GFP**	*Oct4*$^{+/+}$	An eGFP-ires-pacpA cassette containing an Oct4 binding site mutation in the proximal Nanog promoter was inserted at the AUG codon of *Nanog* in E14Tg2a cells.
**D7A3 PE**	*Oct4*$^{+/+}$	Parietal endoderm (*Lif*$^{-/-}$) cell line

Genetically engineered cell lines used in this study; unless otherwise indicated all lines are ES cells, related to all Figures ([1]Ying et al., 2002; [2] Mountford et al., 1994; [3]Niwa et al., 2000; [4]Chambers et al., 2007 (previously referred as TNG); [5]Gilchrist et al., 2003; [6]Wilder et al., 1997)

Table S2. Oligonucleotides, Related to Figures 1, 3, and 6

TARGET	FORWARD SEQUENCE	REVERSE SEQUENCE
Brachyury	CAGCCCACCTACTGGCTCTA	GAGCCTGGGGTGATGGTA
Esrrb	TGAGGGTAACCTTTCCTTGC	ACGACATTCGGTTCAGCAG
FGF4	CCGGTTCTTCGTGGCTATGA	CTTACTGAGGGCCATGAACAT
FGF5	TTGCGACCCAGGAGCTTAAT	CTACGCCTCTTTATTGCAGC
Foxa2	CATCCGACTGGAGCAGCTA	GCGCCCACATAGGATGAC
GFP	AAGTTCATCTGCACCACCG	TCCTTGAAGAAGATGGTGCG
Nanog	CCTCCAGCAGATGCAAGAA	GCTTGCACTTCATCCTTTGG
Nestin	CTGCAGGCCACTGAAAAGT	TTCCAGGATCTGAGCGATCT
Oct4	GTTGGAGAAGGTGGAACCAA	CTCCTTCTGCAGGGCTTTC
Pax6	GTTCCCTGTCCTGTGGACTC	ACCGCCCTTGGTTAAAGTCT
Rex1	CAGCTCCTGCACACAGAAGA	ACTGATCCGCAAACACCTG
Sox17	CACAACGCAGAGCTAAGCAA	CGCTTCTCTGCCAAGGTC
Sox2	GTGTTTGCAAAAAGGGAAAAGT	TCTTTCTCCCAGCCCTAGTCT
TBP	GGGGAGCTGTGATGTGAAGT	CCAGGAAATAATTCTGGCTCA
Tuji	GCGCATCAGCGTATACTACAA	TTCCAAGTCCACCAGAATGG
Wnt3a	AATGGTCTCTCGGAGTTTG	CTTGAGGTGCATGTGACTGG

Sequences of oligonucleotides used in this study, related to Figures 1, 3, and 6

Supplemental Experimental Procedures

Injection of Cells into Blastocysts

GFP-expressing *Oct4*$^{+/-}$ ESCs were obtained by transfection of a CAG driven GFP construct into OKO160 or by targeting *Oct4* in AGFP7 ESCs (Gilchrist et al., 2003). Clones expressing GFP ubiquitously were expanded for 7 days in N2B27 containing recombinant LIF (Sigma, Cat no. L5283). Cells were then trypsininised, replated (again at clonal density) and cultured in N2B27/recombinant LIF for a further 7 days. Cells were then injected into the blastocoel cavity of a host C57/Bl6 host as described (Nagy et al., 2003).

Episomal Supertransfection

Wild-type (E14/T) or Nanog-overexpressing (Nanog/T; EF4 derived (Chambers et al., 2003) ESCs that also express the polyoma large T antigen (E14/T) were transfected with Polyoma *ori*$^{+}$ plasmids using Lipofectamine 2000 (Invitrogen; 11668-019) with 3µg of pPyCAGEVIH, or pPyCAGOct4IH. The next day 5 x 10^4 cells were replated in the presence of hygromycin for 5 days to establish colonies and images captured. The same day, hygromycin-resistant populations were obtained by pooling resistant colonies and the next day transfection of a CAG-Cre plasmid was performed using Lipofectamine 2000 in order to excise the loxP-flanked Nanog ORF. The following day cells were plated at clonal density (60 cells/cm^2), cultured for 6 days and colony morphology and GFP status scored.

Mutagenesis of the Octamer Site in the *Nanog* Promoter

The Nanog-GFP-ires-pac targeting vector (Chambers et al., 2007) was used as a substrate for PCR-based mutagenesis in which an oligonucleotide encompassing the mutation was used to amplify a short section of the promoter delimited by unique

restriction sites. Enzyme digestion and ligation was used to construct the mutant vector.

Southern Blotting

DNA isolation was performed using the DNeasy Blood and Tissue kit (Qiagen catalogue no. 69504). 4 µg of DNA were digested with the *Sex*A1. Reactions were loaded onto a 0.8% TAE gel and electrophoresis was performed at 30V overnight. The next day the gel was uv irradiated, washed in 0.5M Sodium Hydroxide/1M Sodium Chloride for 30 min and 0.5M Tris/3M Sodium Chloride (pH 7.4) for 45 min and blotted onto a Hybond XL membrane. Pre-hybridisation was performed in Perfecthyb (Sigma, H7033) containing salmon sperm DNA and hybridisation was performed in Perfecthyb (Sigma, H7033) with 10 ng of $dCTP^{32}$-labeled probe DNA overnight at 65ºC. Membrane was washed twice in 0.5X SSC 0.1% SDS at 65ºC for a total of 60 min and exposed to Hyperfilm (Amersham).

Flow Cytometry

Cells were analysed using a Becton-Dickinson (Palo Alto, California) FACSCalibur or a Becton-Dickinson Fortessa LSRII. Cells were incubated in cell dissociation buffer (Gibco cat. no. 13151-014) for 5 min or Trypsin for 1 min and resuspended at approximately 10^6/ml in PBS/10% FCS. Anti-SSEA1 antibody (MC-480, developed by D. Solter and B. Knowles, obtained from the Developmental Studies Hybridoma Bank, Department of Biological Sciences, University of Iowa, Iowa City, USA) was added to cells at 1:1,000 dilution of ascites, cells were incubated (4°C,15 min) and washed in PBS/10% FCS. Phycoerythrin-conjugated anti-IgM was then added at 1:1000 (15 min, 4°C).

Intracellular FACS staining was performed as described (Festuccia and Chambers, 2011).

Protein Analysis

Immunofluorescence was performed at clonal density as described (Lowell et al., 2006). Primary antibodies used were: Nanog (Chambers, 2004), Oct4 (C10; Santa Cruz cat. no. sc-5279), β-III tubulin (Tuji; Covance cat. no. MMS-435P), Nestin (Developmental Studies Hybridoma Bank cat. no. Rat-401-S), Esrrb (R&D Systems cat. no. PP-H6707-00), Klf4 (R&D Systems cat. no. AF 3158), phospho-STAT3 (Cell Signalling cat. no, 9145S).

Embryo staining was performed as described (Nichols et al., 2009). Primary antibodies used: Nanog (eBioscience 14-5761-80), Oct4 (C10; Santa Cruz cat. no. sc-5279) or (N19; Santa Cruz cat. no. sc-8628), GFP (Invitrogen A21311).

For immunoblot analysis, $8x10^5$ cells were lysed in 150 µl of 50mM Tris pH 8, 150 mM NaCl, 0.5% NP-40 and treated with Benzonase (Novagen), 20min, 4°C. 40 µg of lysate was then subjected to SDS–PAGE (Laemmli, 1970) and transferred to PVDF membrane (BioRad). Blots were blocked in 10% non-fat dry milk/PBS, 0.01% Tween 20 (1 hour, rt), incubated overnight (4°C) with the primary antibody diluted in 5% non-fat dry milk/PBS/0.01% Tween 20, washed three times in PSB/0.01% Tween 20, incubated with secondary antibody (1 h, rt) in 5% non-fat dry milk/PSB/0.01% Tween 20 followed by three washes in PSB/0.01% Tween 20. Membranes were then developed using Super-Signal West Pico (Pierce) for 5 min (rt) and exposed to Hyperfilm (Amersham). Primary antibodies: anti Nanog (Chambers, 2004) 0.5 µg/ml, anti Oct4 (Santa Cruz sc-5279) 2 µg/ml, anti HDAC2 (Upstate 05-814) 0.1 µg/ml.

For immunoblot of ERK and STAT3 phosphorylation. 1x10^6 ESCs were replated overnight (10cm^2) in GMEMβ/10% FCS/LIF (100 units/ml). The next day, cells were washed and medium was changed to GMEMβ/1% FCS lacking cytokines. The following day cells were changed to GMEMβ for 4 hours before stimulation with 100 units/ml of recombinant LIF (Sigma L5283-10UG) or 10ng/ml of recombinant FGF2 (R&D Systems cat. no 233-FB/CF).

For phospho-STAT3 and phospho-erk immunoblot analysis cells were lysed in 200 µl of sample buffer (Laemmli, 1970), sonicated and microcentrifuged (13,000rpm, 30 mins, 4°C). 15 µl of lysate was then subjected to SDS–PAGE (Laemmli, 1970) and transferred to nitrocellulose membrane (Protran BA83). Blots were processed as above except that blocking was done in 5%BSA/TBS and in subsequent incubations, 5%BSA/TBS was used in place of 10%non-fat dry milk/PBS. Primary antibodies: pSTAT3 (Cell Signalling 9131S) used at 1:1000, β-actin-HRP (AbCam 20272) used at 1:10.000, phospho-ERK (Cell Signalling 9101S) used at 1;1000 and total ERK (Cell Signalling 9102S) used at 1;2000.

RNA Analysis

Total RNA was prepared using the RNeasy mini kit (Qiagen catalogue no. 74106) with DNase (Qiagen catalogue no. 79254) treatment. cDNA was synthesised using a Superscript First Strand Synthesis kit (Invitrogen catalogue no. 12371-019) and PCR performed on a LightCycler 480 (Roche) with cDNA equivalent to 200 ng total RNA. The monocolour hydrolysis probe protocol consisted of denaturation at 95°C for 5 min followed by 45 cycles of 95°C, 10s; 61°C, 10s with a single data acquisition during each extension cycle. Primers for Q-PCR are listed in Supplementary Table 2.

RNA for Microarray analysis was prepared and analysed as in (Festuccia et al., 2012).

Microarray Analysis

Microarray data was processed in R using the *beadarray* (Dunning et al., 2007) and *limma* (Smyth, G.K. 2005) packages. Briefly, we applied a quantile normalization to the raw expression values, removed low-quality probes and used the *limma* algorithms to assess differential expression between all sample groups. We considered genes differentially expressed which were consistently up-regulated or consistently down-regulated (FDR-adjusted p-value ≤ 0.1 and absolute $\log_2$-fold change ≥ $\log_2(1.25)$) in all four *Oct4*$^{+/-}$ versus *Oct4*$^{+/+}$ comparisons (OKO160/E14Tg2A, ZHTc6/E14Tg2A, OKO160/OctGIP and ZHTc6/ OctGIP). For further data integration, we uploaded the microarray analysis results into the web-based GeneProf software (Halbritter et al., 2012). Multiple probes for the same gene were resolved by picking the probe with the lowest p-value in the OKO160/E14Tg2A comparison. Functional enrichment analysis of the candidate genes was performed via the DAVID tool (Huang et al., 2009) using a background control of all genes that were measured on the arrays and considered expressed in at least one of the datasets at hand (n = 18,336).

ChIP-Seq

ESCs were fixed with 1% formaldehyde (10 min, rt) and crosslinking stopped by addition of glycine to 0.2 M. Cells were lysed by incubating in 10 mM Tris-HCl, 0.25% Triton X-100, 10 mM EDTA, 100 mM NaCl (twice, 15min, 4°C). Nuclei were lysed in 50 mM HEPES-KOH, 150 mM NaCl, 2 mM EDTA, 1% Triton X-100, 0.1% deoxycholate, 1% SDS (15 min, 4°C), centrifuged and the chromatin pellet resuspended in 0.1% SDS buffer for shearing on ice to an average size of about 250 bp using a probe sonicator (Branson digital sonifier S-450D). Chromatin extracts were pre-cleared with Protein G Dynal Magnetic Beads (Invitrogen) (2 h, 4°C) and

immunoprecipitated overnight at 4°C using Protein G Dynal Magnetic Beads pre-coupled with antibodies against Oct4 (Santa Cruz N19, sc8628), Nanog (Cosmo Bio RCAB0002P-F) or H3K27me3 (Millipore 07-449). Beads were washed 3x with 0.1% SDS buffer, once with 0.1% SDS/ 0.35M NaCl buffer, once in 10 mM Tris-HCl, 0.25M LiCl, 1 mM EDTA, 0.5% deoxycholate, 0.5% NP-40, and once in TE buffer (10 mM Tris-HCl, 1 mM EDTA). Immunoprecipitated material was eluted from the beads and crosslinks reversed by incubation with pronase for 2 h at 42°C then 6 h at 67°C. DNA was extracted by phenol/chloroform/isoamyl-alcohol followed by chloroform, then precipitated with ethanol and resuspended in TE buffer. ChIP-Seq library was prepared using the ChIP-Seq Sample Preparation Kit (Illumina) and NEBNext® ChIP-Seq Library kit (NEB Biolabs) according to the manufacturer's instructions and sequenced for 36 cycles with the HiSeq 2000 system (Illumina).

Chip Analysis

Reads were aligned against the mm9 reference genome using Bowtie version 0.12.8 (Langmead et al., 2009) with options –m1 –v 2. Peak calling was done using MACS version 1.4.2 (Zhang et al., 2008) with a control data set for *Oct4*$^{+/+}$ ESCs and *Oct4*$^{+/-}$ ESCs. Genome-wide coverage was calculated using Rsamtools (Li et al., 2009). Read counts were normalised by the number of uniquely mapped reads. Fold changes of binding intensities were estimated by the fold change of reads that could be mapped within a 500 bp window around the binding peak. Since the number of reads which are mapped to peaks differs between samples, a normalised fold change was calculated based on the quantile normalised read counts. We selected the top 1500 binding sites with the strongest fold change differences to identify loci with increased Oct4 occupancy in *Oct4*$^{+/-}$ and *Oct4*$^{+/+}$ ESCs. This cut-off corresponds to a fold change cut-off of 2.59 for *Oct4*$^{+/-}$ ESC-specific binding events and 2.2 for *Oct4*$^{+/+}$ ESC-specific binding events. The *Oct4*$^{+/-}$ ESC-specific binding events were associated

with 1122 protein coding genes based on the nearest transcription start site, *Oct4*$^{+/+}$ ESC-specific binding events were associated with 1270 protein coding genes. As the set of shared loci we selected the 7500 peaks that showed the smallest differences in Oct4 binding (fold change between 1.07 and 1/1.07). The set of genes with reduced levels of H3K27me3 in *Oct4*$^{+/-}$ ESC cells corresponds to the 1000 genes with the highest reduction of H3K27me3 levels in a 4000bp window around the transcription start site compared to *Oct4*$^{+/+}$ ESCs. Differentially expressed genes which were used to calculate the overlap with Oct4 binding were selected as described above, but based on the comparison between OKO160 and E14Tg2a cells, to match the cell lines used to obtain the genome-wide binding profiles. Significance of the enrichment of genes with differential binding and differential histone modifications was estimated using Fisher's Exact Test, the total number of genes used for this test was 21,458.
Public data sets were obtained from the European Nucleotide Archive (ENA) (Creyghton et al., 2010).

Data Access

The ChIP-Seq data are accessible at the ArrayExpress Archive under accession number E-MTAB-1617.

The microarray data are accessible at the ArrayExpress Archive under accession number E-MTAB-1619.

Supplemental References

Chambers, I. (2004). Mechanisms and factors in embryonic stem cell self-renewal. Rend Fis Acc Lincei *s.9, v.16*, 83-97.

Chambers, I., Colby, D., Robertson, M., Nichols, J., Lee, S., Tweedie, S., and Smith, A. (2003). Functional expression cloning of Nanog, a pluripotency sustaining factor in embryonic stem cells. Cell *113*, 643-655.

Chambers, I., Silva, J., Colby, D., Nichols, J., Nijmeijer, B., Robertson, M., Vrana, J., Jones, K., Grotewold, L., and Smith, A. (2007). Nanog safeguards pluripotency and mediates germline development. Nature *450*, 1230-1234.

Chen, X., Xu, H., Yuan, P., Fang, F., Huss, M., Vega, V.B., Wong, E., Orlov, Y.L., Zhang, W., Jiang, J., *et al.* (2008). Integration of external signaling pathways with the core transcriptional network in embryonic stem cells. Cell *133*, 1106-1117.

Creyghton, M.P., Cheng, A.W., Welstead, G.G., Kooistra, T., Carey, B.W., Steine, E.J., Hanna, J., Lodato, M.A., Frampton, G.M., Sharp, P.A., *et al.* (2010). Histone H3K27ac separates active from poised enhancers and predicts developmental state. Proceedings of the National Academy of Sciences of the United States of America *107*, 21931-21936.

Dunning, M.J., Smith, M.L., Ritchie, M.E., Tavaré, S. (2007). beadarray: R classes and methods for Illumina bead-based data. Bioinformatics 23, 2183-2184.

Festuccia, N., and Chambers, I. (2011). Quantification of pluripotency transcription factor levels in embryonic stem cells by flow cytometry. Curr Protoc Stem Cell Biol *Chapter 1*, Unit 1B 9.

Gilchrist, D.S., Ure, J., Hook, L., Medvinsky, A. (2003) Labeling of Hematopoietic Stem and Progenitor Cells in Novel Activatable Egfp Reporter Mice. genesis 36, 168-176

Kagey, M.H., Newman, J.J., Bilodeau, S., Zhan, Y., Orlando, D.A., van Berkum, N.L., Ebmeier, C.C., Goossens, J., Rahl, P.B., Levine, S.S., *et al.* (2010). Mediator and cohesin connect gene expression and chromatin architecture. Nature.

Halbritter, F., Vaidya, H.J. and Tomlinson, S.R. (2012). GeneProf: analysis of high-throughput sequencing experiments, Nature Methods 9, *in press.*

Huang, D.W., Sherman, B.T. & Lempicki, R.A. (2009). Systematic and integrative analysis of large gene lists using DAVID Bioinformatics Resources. *Nature Protocols* 4(1), pp. 44-57.

Huang, D.W., Sherman, B.T. & Lempicki, R.A. (2009). Bioinformatics enrichment tools: paths toward the comprehensive functional analysis of large gene lists. *Nucleic Acids Research* 37(1), pp.1-13.

Laemmli, U.K. (1970). Cleavage of structural proteins during the assembly of the head of bacteriophage T4. Nature *227*, 680-685.

Langmead, B., Trapnell, C., Pop, M., and Salzberg, S.L. (2009). Ultrafast and memory-efficient alignment of short DNA sequences to the human genome. Genome biology *10*, R25.

Li, H., Handsaker, B., Wysoker, A., Fennell, T., Ruan, J., Homer, N., Marth, G., Abecasis, G., and Durbin, R. (2009). The Sequence Alignment/Map format and SAMtools. Bioinformatics *25*, 2078-2079.

Lowell, S., Benchoua, A., Heavey, B., and Smith, A.G. (2006). Notch promotes neural lineage entry by pluripotent embryonic stem cells. PLoS Biol *4*, e121.

Mountford, P., Zevnik, B., Duwel, A., Nichols, J., Li, M., Dani, C., Robertson, M., Chambers, I., and Smith, A. (1994). Dicistronic targeting constructs: reporters and modifiers of mammalian gene expression. Proc Natl Acad Sci USA *91*, 4303-4307.
Nagy, A., Gertsenstein, M., Vintersten, K., and Behringer, R. (2003). Manipulating the Mouse Embryo: A Laboratory Manual, 3rd edn (New York, Cold Spring Harbor Press).
Smyth, G.K. (2005). Limma: linear models for microarray data. In Bioinformatics and Computational Biology Solutions using R and Bioconductor., R. Gentleman, V. Carey, S. Dudoit, R. Irizarry, W. Huber, eds. (New York, USA: Springer Verlag), pp. 397-420
Wilder, P. J., Kelly, D., Brigman, K., Peterson, C. L., Nowling, T., Gao, Q. S., McComb, R. D., Capecchi, M. R., and Rizzino, A. (1997). Inactivation of the FGF-4 gene in embryonic stem cells alters the growth and/or the survival of their early differentiated progeny. Dev Biol *192*, 614-629.
Ying, Q. L., Nichols, J., Evans, E. P., and Smith, A. G. (2002). Changing potency by spontaneous fusion. Nature *416*, 545-548.
Zhang, Y., Liu, T., Meyer, C.A., Eeckhoute, J., Johnson, D.S., Bernstein, B.E., Nusbaum, C., Myers, R.M., Brown, M., Li, W., *et al.* (2008). Model-based analysis of ChIP-Seq (MACS). Genome biology *9*, R137.
